# Transcriptomic analysis of *ROS1*+ non-small cell lung cancer reveals an upregulation of nucleotide synthesis and cell adhesion pathways

**DOI:** 10.3389/fonc.2024.1408697

**Published:** 2024-12-16

**Authors:** Marc Terrones, Ken Op de Beeck, Guy Van Camp, Geert Vandeweyer, Ligia Mateiu

**Affiliations:** ^1^ Center of Medical Genetics, University of Antwerp and Antwerp University Hospital, Edegem, Belgium; ^2^ Center for Oncological Research, University of Antwerp and Antwerp University Hospital, Wilrijk, Belgium

**Keywords:** ROS1+ NSCLC, RNA-sequencing, gene co-expression, nucleotide synthesis, cell adhesion, prognostic biomarker

## Abstract

**Introduction:**

The transcriptomic characteristics of *ROS1*+ non-small cell lung cancer (NSCLC) represent a crucial aspect of its tumor biology. These features provide valuable insights into key dysregulated pathways, potentially leading to the discovery of novel targetable alterations or biomarkers.

**Methods:**

From The Cancer Genome Atlas (TCGA) and the Gene Expression Omnibus (GEO) databases, all available *ROS1*+ (n = 10), *ALK*+ (n = 5) and *RET*+ (n = 5) NSCLC tumor and *ROS1*+ cell line (n = 7) RNA-sequencing files were collected. In addition, 10 healthy lung RNA-seq samples were included. Differential gene expression with DESeq2 (R package) and gene co-expression (WGCNA, R package) analyses were performed. Functional annotation was performed through Gene Set Enrichment Analysis (GSEA) using Webgestalt and RNAseqChef, Over-Representation Analysis (ORA) through Enrichr. iRegulon was used to identify enriched transcription factors that regulate a gene co-expression module.

**Results:**

*ROS1*+ NSCLC samples were significantly enriched for the nucleotide synthesis and cell adhesion KEGG pathways compared to *ALK*+ and *RET*+ samples. Moreover, *NOTCH1* was significantly downregulated in *ROS1*+ NSCLC and PD-L1 was weakly expressed. When comparing *ROS1*+ tumor versus cell line transcriptomes, an upregulation of *MYC* and *MET* was found in cell lines together with a significantly decreased expression of *HER3*, *HER4* and *BRAF*. Within *ROS1*-tumors, *GJB2* was overexpressed in the *CD74*- and *CLTC*-*ROS1*+ subgroups. The differential expression of *IL20RB* and *GJB2* in cell lines was confirmed through RT-qPCR. Finally, the gene co-expression analysis unveils a gene cluster involving cell cycle-related genes which significantly correlates with the disease stage of patients. In addition, we propose *TFDP1* and *ISL1* as key *ROS1*-specific transcription factors.

**Conclusion:**

This study highlights cell adhesion and nucleotide synthesis as crucial signatures in *ROS1*+ NSCLC. The upregulation of *GJB2* may serve as a prognostic biomarker, along with *IL20RB*, a known mediator of bone metastases. Furthermore, *TDFP1* and *ISL1* were identified as relevant transcription factors that could potentially regulate the biological processes in *ROS1*-rearranged NSCLC.

## 1 Introduction

*ROS1*+ non-small cell lung cancer (NSCLC) is a molecular subgroup of malignancies which account for approximately 2% of newly diagnosed lung cancer cases every year (1, 2). Chromosomal rearrangements that involve the 6q22 locus harboring *ROS1* result in the formation of oncogenic fusion proteins. Thus, *ROS1*+ NSCLC belongs to the category of oncogene-addicted tumors like *ALK*+, *RET*+ and *NTRK*+ NSCLC, among others (3, 4). The overexpression of a constitutively activated tyrosine kinase-containing fusion protein promotes cell growth, proliferation and migration through the stimulation of the MAPK, PI3K/mTOR and JAK/STAT signaling pathways (1). Oncogene-addicted lung adenocarcinomas are predominantly diagnosed in young, non-smoker patients. They present a low tumor mutational burden (TMB) and show a poor response towards immune checkpoint inhibitors (5). Concerning the treatment of *ROS1*+ NSCLC, tyrosine kinase inhibitors (TKIs) are the most effective targeted therapies in first line. However, resistance is observed in the majority of patients after a certain period of treatment, caused by intrinsic (e.g. kinase point mutations that prevent inhibitor binding) or extrinsic resistance mechanisms, such as histological transformation (e.g. to small cell lung cancer) or the activation of bypass signaling cascades such as EGFR or c-MET (6–9). Although last-generation TKIs such as repotrectinib and the investigational NVL-520 have shown potent activity against the aggressive ROS1 kinase domain point mutations like G2032R (10, 11), heavily pre-treated patients presenting either extrinsic resistance or brain, bone or liver metastases represent a clinical challenge. Unless other targetable alterations are present driving resistance, patients in this setting rely on chemotherapy-based regimes, with a limited benefit (12). In addition, a remarkable heterogeneity in disease outcomes and metastatic patterns is typically observed among patients, highlighting the need for a deeper understanding of the *ROS1*+ NSCLC tumor biology (13, 14).

Intriguingly, the biological processes defining *ROS1*+ NSCLC beyond the tyrosine kinase-mediated signaling remain largely underexplored. This pathway canonically induces the neoplastic transformation of alveolar type II cells. However, the dysregulation of molecular processes not directly orchestrated by the ROS1 signaling requires further elucidation. It is known that *ROS1* rearrangements, due to their pro-tumorigenic role, are mainly mutually exclusive with alterations affecting homologous kinases such as ALK, RET and NTRK. Despite converging in the same cellular signaling axes, each oncogenic fusion type results in different disease trajectories. For instance, *ALK*+ and *RET*+ NSCLC patients are at higher risk of developing brain metastases compared to *ROS1*+ NSCLC during treatment (15). Moreover, the presence of additional mutations that might explain the tumor evolution, such as tumor-suppressor inactivating variants or copy number gains of oncogenes, has not been fully characterized within the *ROS1*+ NSCLC setting. Although keystone findings in the initiation and evolution of lung adenocarcinoma have been recently published, little attention is paid to *ROS1*-driven tumors (16). This can be explained by the low prevalence of *ROS1* rearrangements across NSCLC patients; which restricts the access to the substantial amount of tumor specimens required to conduct solid studies.

The culprits behind the heterogeneous disease outcomes, poor response to immune checkpoint inhibitors and metastases development of *ROS1*+ NSCLC have not been highlighted yet. Nevertheless, developmental signaling pathways such as the Notch and the Sonic Hedgehog (Shh) pathways are widely known to be aberrantly activated in NSCLC, and co-exist with driver oncogene mutations (17–21). Importantly, Notch signaling orchestrates the epithelial-to-mesenchymal transition (EMT) of NSCLC cells, a critical process at the initial phase of metastasis and drug resistance (22). On top of that, non-canonical oncogenic signaling pathways have lately drawn the attention to the lung cancer field, as exemplified by the SPP1 (osteopontin/phosphoprotein 1) pathway (23). A study by Liu et al. unveiled through single cell RNA-sequencing the pro-tumorigenic role of *GJB2* in LUAD (24). This gene encodes the gap junction beta-2 protein belonging to the connexin family, which was described to modulate intercellular communication and extracellular matrix remodeling. Hence, assessing the expression levels of genes involved in the Notch and Shh pathways together with *GJB2* will shed light on the *ROS1*+ NSCLC pathomechanisms.

Some specific insights into *ROS1*+ tumor evolution were provided in a recent study by Neel et al., demonstrating that the subcellular localization determined by the *ROS1* fusion partner gene modulates the downstream signaling pathway that will be activated (25). This phenomenon will likely be reflected at a gene expression level. Besides contrasting these findings using publicly available data, in this study we hypothesized that *ROS1*+ NSCLC is defined by specific transcriptomic signatures compared to other oncogene-driven tumors like *ALK*+ or *RET*+ lung adenocarcinomas. In parallel to the deeper exploration of the biology of *ROS1*+ NSCLC, the second goal of this exploratory analysis consists in identifying candidate genes whose dysregulated expression in a *ROS1*+-specific manner might be considered as novel therapeutic targets or biomarkers.

## 2 Methods

### 2.1 Obtaining gene expression data

Gene expression RNA-seq data files were obtained from publicly available databases Gene Expression Omnibus (GEO) (fastq) and The Cancer Genome Atlas (TCGA) (raw gene counts) after selecting for *ROS1*-fusion containing lung cancer RNA-seq samples. The raw read counts for GEO samples were acquired adhering to the specifications outlined in the published GDC mRNA quantification analysis pipeline, which was also utilized for TCGA samples (GRCh38.d1.vd1_gencode.v36 GDC reference genome, STAR 2.7.5c).

### 2.2 Patient-derived cell lines

HCC-78 cells were obtained from the German Collection of Microorganisms and Cell Cultures GmbH (DSMZ, Germany). CUTO-28, CUTO-37, CUTO-38 and CUTO-27 were kindly provided by Prof. Dr. Robert C. Doebele (Division of Medical Oncology, University of Colorado School of Medicine, Anschutz Medical Campus). Cells were cultured in RPMI 1640 medium supplemented with 10% fetal bovine serum (FBS) and L-glutamine 1% v/v; at 37°C, 5% CO_2_ in a humidified incubator.

### 2.3 RT-qPCR

To validate the chosen significant differentially expressed genes (DEGs), 2 μg of total RNA extracted with QIAgen RNeasy RNA isolation kit was employed to synthesize the complementary DNA (cDNA) using the SuperScriptTM III First-Strand Synthesis kit (Thermo Fisher, cat. # 18080051). The following primers were designed to assess the expression levels of the genes (5’➔3’): *GJB2* (FWD: TGGTGGACCTACACAAGCA, REV: TGGAGAAGCCGTCGTACAT), *IL20RB* (FWD: CTGAAGGTCCTGAGTGTGATG, REV: GAGGTCTGTGAGCCCAATG), *GAPDH* (FWD: TGCACCACCAACTGCTTAGC, REV:

GGCATGGACTGTGGTCATGAG), *HPRT1* (FWD: TGACACTGGCAAAACAATGCA, REV:

GGTCCTTTTCACCAGCAAGCT) and *YWHAZ* (FWD: CGAAGCTGAAGCAGGAGAAG, REV: TTTGTGGGACAGCATGGATG). The quantitative PCR was performed using the SYBR Green Master Mix 2x (Eurogentec, cat. # RT-SN2X-03+) in a Bio-Rad CFX96 real-time PCR system. Data was analyzed with the Bio-Rad CFX Maestro software (Bio-Rad v2.3) and qbasePLUS (Biogazelle). Gene expression levels are reported as calibrated and normalized relative quantities (CNRQ) ± standard error (SE) obtained from the gene normalization considering *GAPDH*, *HPRT1* and *YWHAZ* as reference target genes.

### 2.4 Statistical methods

#### 2.4.1 Differential gene expression

The read counts associated with protein coding genes from all samples were consolidated into a matrix, serving as input for the DESeq2, R package (26). Gene counts related to Y chromosome, mitochondrial, and ribosomal RNA were excluded from subsequent analysis. In the differential gene expression analysis, the batch effect correction was applied using the removeBatchEffect function from the limma R package (27). Multiple contrasts were defined to assess differential expression across various groups within the study (**Supplementary File 1**). In each comparison, genes with a p-value less than 0.05, following multiple testing correction using the Benjamini-Hochberg (BH) method, were considered to be statistically significant and thus identified as differentially expressed. The cut-offs for the BH method include log_2_(fold change) = |2| and false-discovery rate (FDR) < 0.05. The clustering of the cell line samples was performed through the K-means method. K=7 was chosen given that 7 different cell lines were analyzed and the three biological replicates of each sample clustered together, as verified upon the DESeq2 analysis. Correlation between *ROS1* expression and oncogenes of interest across *ROS1*+ tumor samples was calculated using the Spearman rank method in RNAseqChef (28). Regarding the RT-qPCR experiments, technical triplicates were used per each reaction and every RT-qPCR was performed twice. Differences in *GJB2* and *IL20RB* expression between cell lines were assessed using 1-way ANOVA, a Bonferroni correction for multiple comparisons and α=0.05 in GraphPad Prism v9.

#### 2.4.2 Gene co-expression

The investigation of dysregulated genes involved the utilization of Weighted Gene Co-expression Network Analysis (WGCNA, R package) to identify co-expressed modules (i.e. clusters of genes exhibiting similar expression patterns across samples) and hub genes (i.e. genes playing a central and highly connected role within a co-expression module) (29). It is based on the assumption that highly correlated genes within a module (cluster) are involved in common biological processes. For this analysis, we selected the 70% most variable genes from the gene expression data, after filtering, normalization and batch correction. Then, a signed correlation matrix was created by calculating the biweight midcorrelation (a robust alternative to the Pearson correlation) across all gene pairs. This adjacency matrix was obtained using a soft threshold power of 8 to establish a scale-free topology. The topological overlap measure (TOM) was then computed for all genes, considering both direct pairwise correlations and shared correlations with other genes. Unsupervised clustering of genes in the hierarchical cluster tree, based on a dissimilarity threshold of 1-TOM, resulted in the formation of gene modules. In this study, the minimum module size was defined as 100 genes, and the module-merging cut height was set at 0.3.

#### 2.4.3 Functional annotation

The functional characterization of the differentially expressed genes was carried out through different methods. RNAseqChef was used to perform a gene set enrichment analysis (GSEA) and over-representation analysis (ORA) together with Webgestalt and Enrichr (30, 31).

#### 2.4.4 Kaplan-Meier survival curves

Survival data obtained from the lung adenocarcinoma (LUAD) cohort available at TCGA database was plotted with GEPIA2 (32). All the subtypes were included in the cohort: proximal inflammatory (PI), proximal proliferative (PP) and terminal respiratory unit (TRU).

## 3 Results

### 3.1 Patient characteristics

With regard to the *ROS1*+ patient characteristics shown in **Table 1**, among samples gathered in this study (n=10), the median age at diagnosis was 63 years-old and both sexes are represented in a balanced manner, with 50% males and 50% females respectively. Diverse ethnicities are also present in the study, with 50% of Caucasian (5/10), 33% of Asian (3/10) and 10% of African-American (1/10) descent. Concerning disease stage, half of the sequenced samples were categorized as a IIB (5/10), followed by stage IIIA and IB in equal proportions each (2/10) and only one sample (1%) was collected at stage IV (1/10). Finally, previous treatment received by the patients was not reported for all samples. Within the available annotations, chemotherapy alone or in combination with radiotherapy or immunotherapy was the most common approach (3/10). Only one patient received radiation, immuno- and chemotherapy previously.

**Table 1:** Characteristics of the samples retrieved from GEO and TCGA.

Sample	GEO Number	Sequenced material	Oncogenic fusion	Age	Sex	Ethnicity	Disease stage		Previous treatment
S39	GSM993681	Tumor + normal	*CD74-ROS1*	58	M	Asian	IIB	n/a	
S9	GSM993651	Tumor + normal	*CCDC6-ROS1*	69	M	Asian	IIB	n/a	
S48	GSM993690	Tumor + normal	*SLC34A2-ROS1*	48	F	Asian	IB	n/a	
TCGA-64-1680-01	n/a	Tumor	*CD74-ROS1*	63	M	Caucasian	IV	RT	
TCGA-86-8278-01	n/a	Tumor	*CD74-ROS1*	63	F	Caucasian	IIB	RT, CT and TKI	
TCGA-44-2665-01	n/a	Tumor	*CLTC-ROS1*	55	F	Caucasian	IIB	CT and IT	
TCGA-55-6986-01	n/a	Tumor	*EZR-ROS1*	74	F	Caucasian	IB	None	
TCGA-NJ-A7XG-01	n/a	Tumor	*EZR-ROS1*	49	M	African American	IIIA	CT	
TCGA-05-4426-01	n/a	Tumor	*SLC34A2-ROS1*	71	M	n/a	IB	n/a	
TCGA-62-A46Y-01	n/a	Tumor	*SLC34A2-ROS1*	70	F	Caucasian	IIIA	RT and CT	
S26	GSM993668	Tumor + normal	*EML4-ALK*	70	F	Asian	IB	n/a	
TCGA-50-8460-01	n/a	Tumor	*EML4-ALK*	74	M	Caucasian	IA	RT	
TCGA-67-6215-01	n/a	Tumor	*EML4-ALK*	52	F	Caucasian	IB	CT	
TCGA-78-7163-01	n/a	Tumor	*EML4-ALK*	60	M	Caucasian	n/a	n/a	
TCGA-86-A4P8-01	n/a	Tumor	*EML4-ALK*	59	F	Caucasian	n/a	n/a	
S2	GSM993645	Tumor + normal	*KIF5B-RET*	62	M	Asian	IIIA	n/a	
S6	GSM993649	Normal	*KIF5B-RET*	58	M	Asian	IA	n/a	
S42	GSM993684	Normal	*KIF5B-RET*	62	F	Asian	IIIB	n/a	
TCGA-75-6203-01	n/a	Tumor	*CCDC6-RET*	n/a	F	n/a	IIIA	n/a	
TCGA-55-6543-01	n/a	Tumor	*TRIM33-RET*	n/a	n/a	n/a	IA	n/a	
TCGA-55-8616-11A	n/a	Normal	–	58	F	Caucasian	n/a	n/a	
TCGA-50-5931-11A	n/a	Normal	–	75	F	Caucasian	n/a	n/a	
TCGA-55-1592-11A	n/a	Normal	–	n/a	M	Caucasian	n/a	n/a	
TCGA-55-6968-11A	n/a	Normal	–	61	M	Caucasian	n/a	n/a	
TCGA-38-4632-11A	n/a	Normal	–	42	M	African American	n/a	n/a	
CUTO23	GSM7675355-57	Cell line	*CD74-ROS1*	n/a	n/a	n/a	n/a	n/a	
CUTO27	GSM7675361-63	Cell line	*CD74-ROS1*	n/a	n/a	n/a	n/a	n/a	
CUTO28	GSM7675367-69	Cell line	*TPM3-ROS1*	n/a	n/a	n/a	n/a	n/a	
CUTO33	GSM7675373-75	Cell line	*CD74-ROS1*	n/a	n/a	n/a	n/a	n/a	
CUTO37	GSM7675379-81	Cell line	*CD74-ROS1*	n/a	n/a	n/a	n/a	n/a	
CUTO38	GSM7675386-88	Cell line	*CD74-ROS1*	n/a	n/a	n/a	n/a	n/a	
HCC78	GSM7675391-93	Cell line	*SLC34A2-ROS1*	65	M	n/a	n/a	n/a	

M, male; F, female; RT, radiotherapy; CT, chemotherapy; TKI, tyrosine kinase inhibitor; n/a, not available.

### 3.2 Nucleotide synthesis and cell adhesion pathways are enriched signatures in *ROS1*+ NSCLC

**Figure 1:**
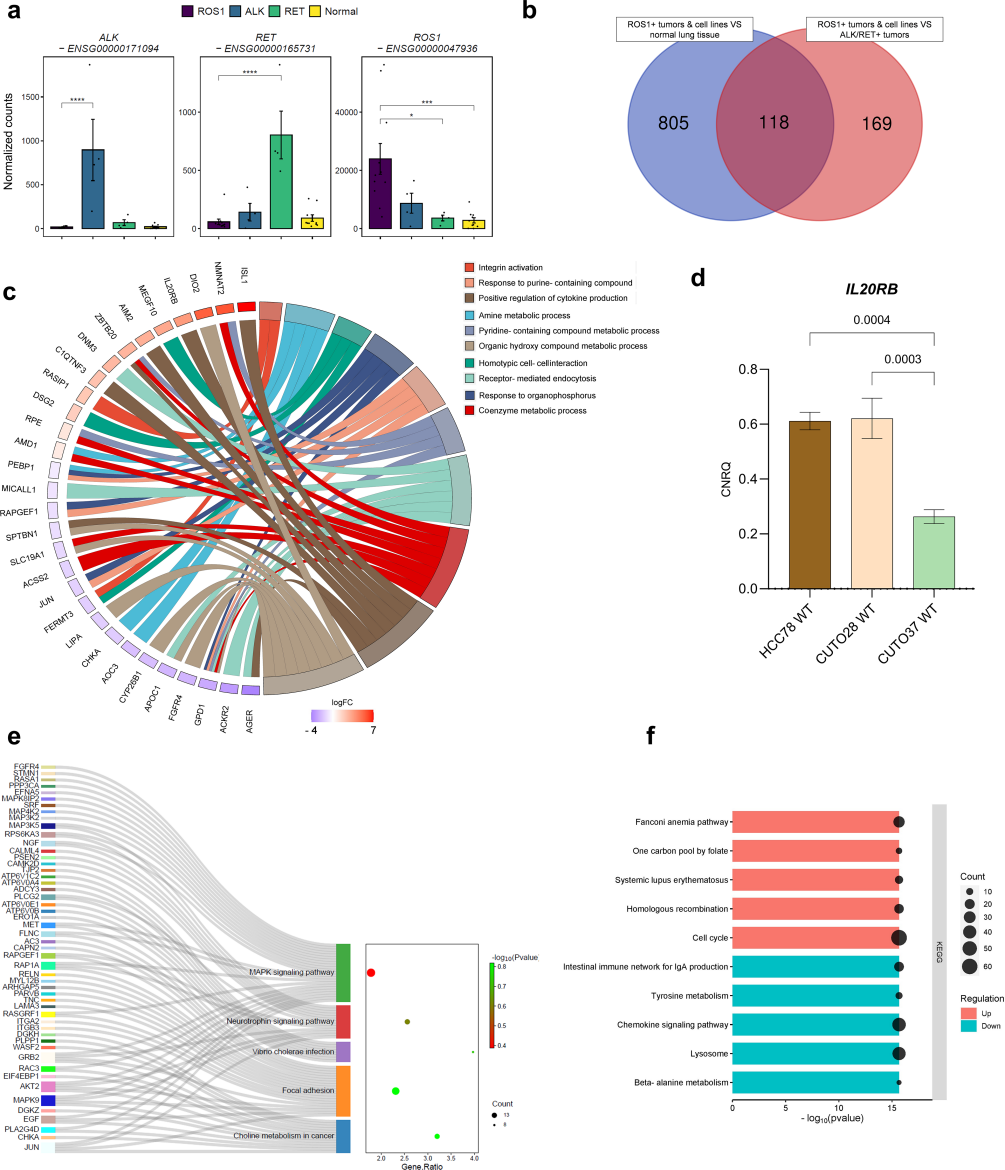
*ROS1*+ NSCLC signature. **(A)** Gene expression levels of the oncogenic kinases in *ALK*+, *RET*+, *ROS1*+ tumors and normal lung tissue. **(B)** Venn diagram reflecting the significant differentially expressed genes (DEGs) between two comparisons: (1) *ROS1*+ tumor specimens and cell lines versus normal lung tissue and (2) *ROS1*+ tumor specimens and cell lines versus *ALK*+ and *RET*+ tumor samples. **(C)** Circos plot reflecting the top 10 enriched biological processes in *ROS1*+ NSCLC. **(D)** *IL20RB* mRNA levels in HCC-78, CUTO-28 and CUTO-37 cell lines expressed as calibrated and normalized relative mRNA quantity (CNRQ) ± SEM. A 1-way ANOVA test was performed considering a Bonferroni correction and a chosen α = 0.05. **(E)** Over-representation analysis (ORA) depicting the key enriched pathways in *ROS1*+ tumor samples and cell lines versus normal lung tissue. **(F)** GSEA summarizing the dysregulated KEGG pathways in *ROS1*+ NSCLC compared to normal lung tissue and *ALK*+/*RET*+ tumor specimens.

To begin with, we sought to confirm the overexpression of the rearranged tyrosine kinase in each of the three tumor types under investigation (**Figure 1A**). While oncogene overexpression was evident across all subtypes, statistical significance was achieved in the following comparisons: *ALK*+ vs *ROS1*+ tumors (p < 0.001), *RET*+ vs *ROS1*+ (p < 0.001), *ROS1*+ vs *RET*+ tumors (p = 0.01), and *ROS1*+ vs normal lung tissue (p < 0.001).

Next, in an effort to profile a *ROS1*+ specific expression signature, transcriptomes of *ROS1*+ tumor samples and *ROS1*+ patient-derived cell lines were compared to normal lung tissue or *ALK*/*RET*+ NSCLC specimens. The number of overlapping significant differentially expressed genes in both contrasts are shown in **Figure 1B**. 118 genes were differentially expressed in *ROS1+* compared to both normal tissue and non-*ROS1*+ NSCLC. 805 genes were found to be specifically and significantly dysregulated in *ROS1*+ samples compared to normal lung tissue. In addition, 169 specifically and significantly differentially expressed genes (DEGs) were obtained from the comparison between *ROS1* and *ALK*/*RET*-driven tumors. We then proceeded with an over-representation analysis (ORA) for the 118 *ROS1*+ specific DEGs to determine which biological functions were enriched in *ROS1*-rearranged tumors and cell lines based on the overlapping 118 gene group. The top 10 enriched categories and the log2(fold change) of the genes associated with each biological function are illustrated in **Figure 1C**. Firstly, metabolic processes related to nucleotide synthesis, such as the response to purine-containing compound, amine or pyridine-containing metabolic process and the response to organophosphorus, were the most enriched. The *ROS1*-specific overexpression of *NMNAT2*, an enzyme involved in nicotinamide adenine dinucleotide (NAD) synthesis, and *ISL1*, a crucial transcription factor regulating glycolysis and tumorigenesis, highlight the metabolic dependencies of *ROS1*+ tumor specimens and cell lines. Moreover, several significantly overexpressed genes, including *IL20RB*, were associated with the positive regulation of cytokine production. Considering the role of *IL20RB* in promoting bone metastasis in lung adenocarcinoma (33), we selected this gene for further validation. RT-qPCR was performed to confirm the expression of *IL20RB* in cell lines, as shown in **Figure 1D**. Furthermore, the analysis revealed an overrepresentation of biological processes related to cell adhesion, including integrin activation (characterized by *RASIP1* overexpression), homotypic cell-cell interaction, and receptor-mediated endocytosis.

To obtain a more comprehensive understanding of the gene expression landscape, we explored the primary differences between *ROS1*+ NSCLC samples and cell lines compared to normal lung tissue. This approach aimed to highlight the crucial pathways that may contribute to the malignant transformation process in this specific tumor subtype. For this purpose, we carried out an over-representation analysis (ORA). As shown in **Figure 1E**, the MAPK signaling pathway (Gene ratio = 1.78, p = 0.4) and the focal adhesion KEGG pathways (GR = 2.31, p = 0.15) contained the highest amount of the significant DEGs. In addition, the choline metabolism in cancer (GR = 3.2, p = 0.15), the neurotrophin signaling pathway (GR = 2.56, p = 0.24) and the *Vibrio cholerae* infection were also over-represented categories (GR = 3.97, p = 0.18). To complement the initial findings, we expanded the GSEA-based analysis to identify significantly upregulated or downregulated pathways in *ROS1*+ lung adenocarcinomas and cell lines in comparison to *ALK*/*RET*+ tumor samples (**Figure 1F**). Interestingly, the analysis revealed an upregulation of gene sets associated with the cell cycle and homologous recombination, accompanied by a downregulation of gene sets related to tyrosine and beta-alanine metabolism, lysosome function, and the chemokine signaling pathway (all p-values < 2e^-16^).

### 3.3 Inflammation-related pathways differentiate *ROS1*+ from *ALK*+ and *RET*+ NSCLC

**Figure 2:**
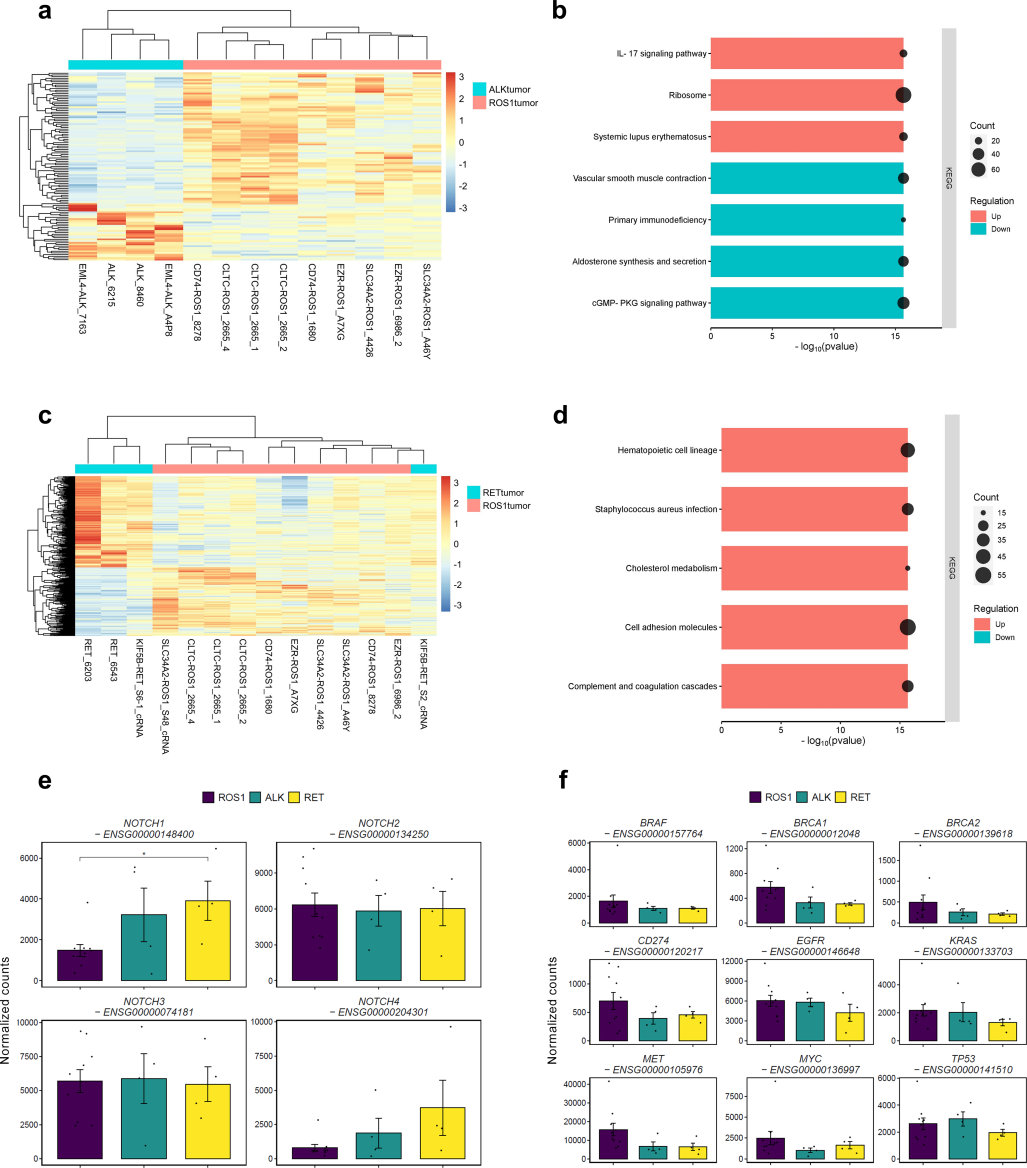
Differences between *ROS1*+ and *ALK*+/*RET*+ tumors. **(A)** Clustergram reflecting the differences between *ROS1*+ and *ALK*+ tumor specimens. **(B)** GSEA resulting from the significantly DEGs between *ROS1*+ and *ALK*+ tumors. **(C)** Clustergram reflecting the differences between *ROS1*+ and *RET*+ tumor specimens. **(D)** GSEA resulting from the significantly DEGs between *ROS1*+ and *RET*+ tumors. **(E)** Gene expression levels of the *NOTCH* family and **(F)** canonical tumor-suppressor genes and oncogenes.

Whole tumor transcriptome analysis enables accurate profiling of both the tumor cells and the stromal compartment, which comprise a heterogeneous population of cells that work together to enhance the fitness of the tumor cells. Therefore, directly comparing *ROS1*+ lung adenocarcinomas with *ALK*+ or *RET*+ tumors, while excluding cell lines, provides further insights into the tumor microenvironment. **Figure 2A** shows the heatmap resulting from clustering *ROS1*+ and *ALK*+ tumor transcriptomes based on the significant DEGs between the two groups. Samples clustered together according to their driver mutation, indicating an oncogene-dependent modulation of the gene expression profile. Subsequent GSEA revealed a significant upregulation of the interleukin-17 signaling pathway, the ribosome signature and the systemic lupus erythematosus KEGG pathways in *ROS1*+ tumors compared to *ALK*+ specimens (**Figure 2B**). In addition, downregulation of the cGMP-PKG signaling pathway, the aldosterone synthesis and secretion signature together with primary immunodeficiency and vascular smooth muscle contraction gene sets was observed in *ROS1*+ vs *ALK*+ tumors. Thus, both up and downregulated gene sets implicate differences concerning inflammatory and protein synthesis pathways. Similarly, *ROS1*+ and *RET*+ specimens were collated, revealing a mixed clustering pattern (**Figure 2C**). In this case, three out of the four *RET*+ samples grouped together whilst one shared higher similarity with *ROS1*+ samples. GSEA unveiled significant upregulation of immune response, hematopoietic cell lineage, cell adhesion and cholesterol metabolism pathways in *ROS1*-rearranged tumors. Interestingly, no significantly upregulated gene sets were observed in *RET*+ specimens (**Figure 2D**).

We explored the impact of the Notch signaling pathway within *ROS1*+ NSCLC, as it is known to exert a pro-tumorigenic role in NSCLC (**Figure 2E**). Although no genes regulated through the Notch signaling pathway were significantly dysregulated, we found that *NOTCH1* expression decreased significantly in *ROS1*+ tumor samples compared to *RET*+ specimens (p=0.04). With regard to the other members of the *NOTCH* gene family, *NOTCH2* and *NOTCH3* reflected similar expression trends across tumor types. Interestingly, *NOTCH4* exhibited a modest expression in *ROS1*+ tumors compared to *ALK*+ and *RET*+ specimens, although the difference did not reach statistical significance. Considering the role of the Notch pathway in promoting epithelial-to-mesenchymal transition (EMT), we conducted a comprehensive analysis to explore the association between *NOTCH1* expression and the metastatic characteristics of lung adenocarcinoma (LUAD) patients across multiple studies. As illustrated in **Supplementary Figure 3A**, the group exhibiting low *NOTCH1* expression levels comprised a significantly higher proportion of individuals with TNM stage M0 (p=0.0021), suggesting an absence of metastatic activity in the tumor. To extend our understanding of the patterns of metastatic progression, we retrieved the clinical details of *ROS1*+, *ALK*+ and *RET*+ NSCLC patients from the “Metastatic NSCLC study” by Jee et al. (34). **Supplementary Figure 3B** shows that *ROS1*+ NSCLC patients presented lower rates of extrathoracic metastases, being the central nervous system (CNS) and liver the most common sites. Contrarily, *ALK*+ and *RET*+ NSCLC patients were diagnosed with bone and soft tissue metastatic lesions as well as CNS and liver. Thus, higher *NOTCH1* expression in *RET*+ specimens might be related to the EMT-promoting effect and enhanced metastatic disease in *RET*+ NSCLC patients.

In addition, changes in expression of canonical tumor-suppressor genes such as *BRCA1*, *BRCA2* and *TP53* as well as oncogenes like *BRAF*, *EGFR*, *KRAS*, *MET* and *MYC* were assessed (**Figure 2F**). No significant differences were observed between the various tumor types, suggesting that the expression of these genes is not dependent on the specific rearrangement. Furthermore, we investigated the expression levels of *CD274*, a gene that encodes the programmed death-ligand 1 (PD-L1), a cell surface protein expressed by neoplastic cells that interacts with its receptor, programmed cell death protein 1 (PD-1). The latter is present in activated T, natural killer (NK) and B lymphocytes, macrophages, dendritic cells (DCs) and monocytes. The interaction between PD-1 and PD-L1 leads to the suppression of cellular immunity against tumor cells. Across the three tumor types, a relatively low level of normalized *CD274* counts was observed, with no significant differences detected. This finding supports the classification of *ROS1*+, *ALK*+, and *RET*+ NSCLC as “cold tumors,” which is consistent with the modest benefit of immunotherapy observed in these patient subsets (35, 36).

### 3.4 Differences between *ROS1*+ specimens and tumor-derived cell lines comprise cell cycle, DNA repair and inflammation pathways

**Figure 3:**
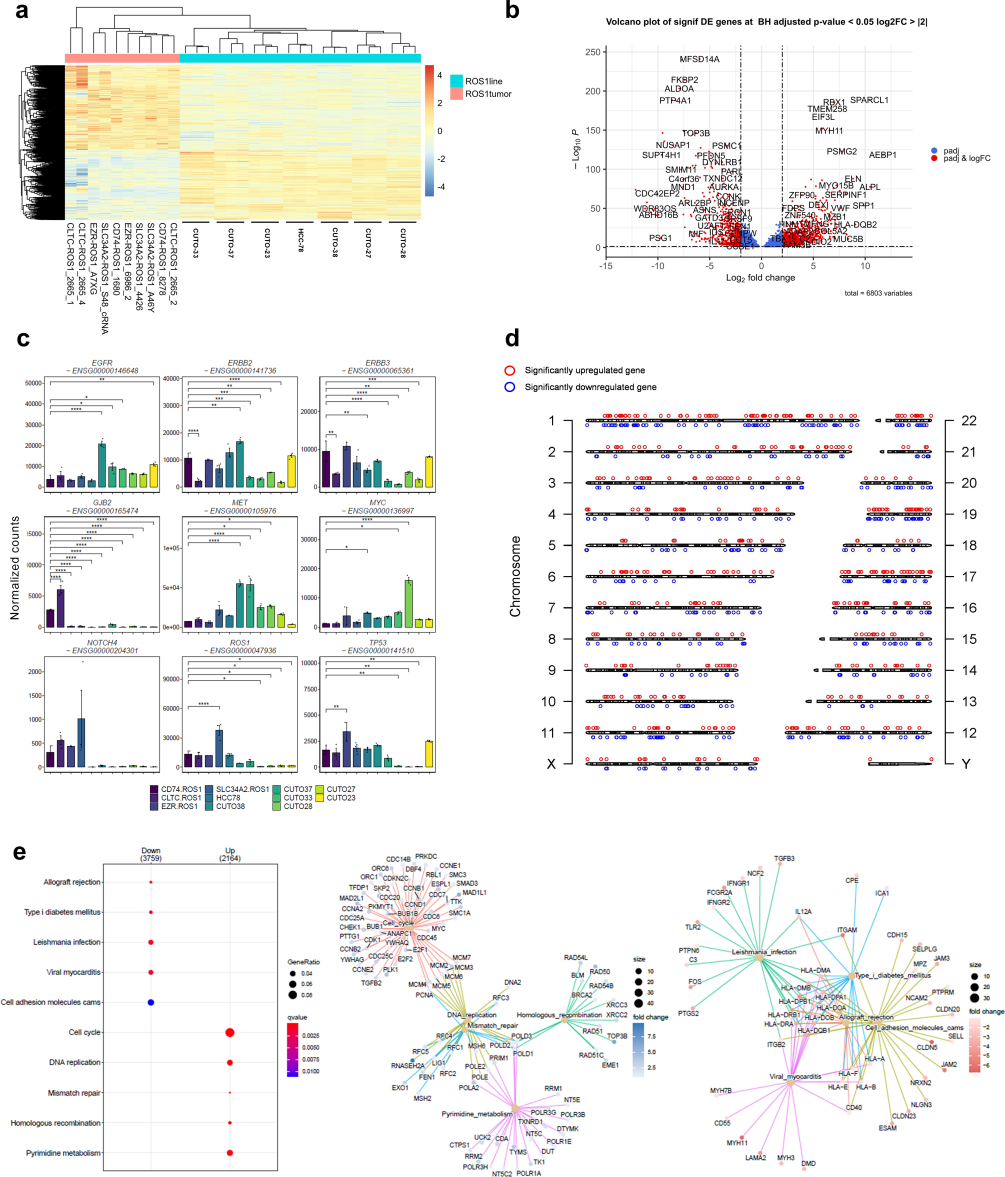
Transcriptomic differences between *ROS1*+ tumors and patient-derived cell lines. **(A)** Clustergram highlighting the differences between *ROS1*+ tumor specimens and patient-derived cell lines. **(B)** Volcano plot containing more than 6,000 DEGs. **(C)** Differential expression of relevant oncogenes and tumor-suppressor genes across *ROS1*+ NSCLC tumor subtypes and patient-derived cell lines. **(D)** Distribution across chromosomes of the top 500 significant up- and downregulated genes between tumor specimens and cell lines. **(E)** GSEA elucidating the KEGG pathways up- or down-regulated in patient-derived cell lines versus tumor specimens.

Given that both *ROS1*+ NSCLC tumor specimens and *ROS1*+ patient-derived cell lines were used in this study, evaluating the key transcriptomic differences between tumor and cell line samples is particularly important. As shown in **Figure 3A**, CUTO and HCC-78 cell lines cluster separately from *ROS1*+ NSCLC tumor samples. This remarkable difference is reflected by the 6,803 significantly DEGs. Interestingly, the HCC-78 cell line gene expression pattern clustered between the one of CUTO-23 and CUTO-38 cell lines, indicating that HCC-78 cells share common transcriptomic traits with CUTO cell lines. Among the significant DEGs shown in in **Figure 3B**, tumor specimens overexpressed genes like *MUC5B*, encoding for the glycoprotein mucin, *AEBP1 (AE binding protein 1)*, *SPARCL1 (SPARC-like protein 1), RBX1 (ring box-1) and ELN (elastin).* In contrast, genes such as *ALDOA (aldolase A), U2AF1 (U2 small nucleolar RNA auxiliary factor 1), MFSD14A (major facilitator superfamily domain containing 14A) and FKBP2 (FKBP prolyl isomerase 2)* were upregulated in patient-derived cell lines.

In order to showcase the representativity of the patient-derived cell lines used in the study, special attention was paid to genes involved in extrinsic resistance mechanisms towards TKIs described in *ROS1*+ NSCLC patients (**Figure 3C**). The statistical tests were performed relatively to *CD74-ROS1* tumor specimens given that it constitutes the most frequent rearrangement subtype. *EGFR* was significantly overexpressed in CUTO-38 cells (p < 0.001), CUTO-37 (p = 0.012), CUTO-33 (p = 0.04) and CUTO-23 (p = 0.003). *ERBB2*, whose product is HER2, was significantly downregulated in *CLTC-ROS1* tumors (p < 0.001), CUTO-37, CUTO-28, CUTO-27 and CUTO-33 (p < 0.001). Contrarily, it was upregulated in CUTO38 cells (p = 0.001). A similar trend was observed in *ERBB3* expression, except for a significant downregulation in HCC-78 cells (p = 0.006) instead of an overexpression in CUTO-38 cells. *GJB2* was clearly expressed in *CD74*- and *CLTC*-*ROS1* tumors, being significantly higher in the latter case (p < 0.001). In contrast, this gene was practically not expressed in the remaining tumor subtypes and cell lines. In parallel, *MET* was upregulated in CUTO38 and CUTO-37 (p < 0.001) together with CUTO-33 (p = 0.024) and CUTO28 (p = 0.011). *MYC* was overexpressed in HCC-78 cells (p = 0.021), CUTO33 (p = 0.017) and CUTO28 (p < 0.001). No differences were detected concerning *NOTCH4* expression. However, the endogenous *ROS1* levels varied across samples. *SLC34A2-ROS1* tumors reflected an upregulation (p < 0.001) whilst CUTO-33, CUTO-28, CUTO-27 and CUTO-23 showed lower *ROS1* levels (p = 0.01). Finally, *TP53* was upregulated in *EZR-ROS1* tumors (p<0.001) and downregulated in CUTO33 (p = 0.007), CUTO-28 (p = 0.004) and CUTO-27 cells (p = 0.005).

To investigate whether specific genomic regions contained a notable concentration of differentially expressed genes (DEGs), the top 500 significant DEGs were mapped to their corresponding locations on the genome (**Figure 3D**). This visualization aimed to provide a graphical representation of the DEG distribution. The analysis revealed that the distribution of these genes was relatively uniform across the genome, with the exception of chromosome 7, which exhibited a higher density of DEGs compared to other chromosomes. GSEA that included the 6,803 DEGs and using the “chromosomalLocation” function revealed a significant enrichment of overexpressed genes in cell lines located in the cytogenetic band chr7p22.3 (**Supplementary Figure 1**). Interestingly, copy number gains involving this genomic region known to harbor oncogenes such as *UNCX, FAM20C, MAD1L1* and *PDGFA* have been reported during the immortalization process of patient-derived small cell lung cancer lines (37).

GSEA identified pathways whose activation differed between tumor samples and cell lines (**Figure 3E**). As expected, immune response-related gene sets like allograft rejection, *Leishmania* infection and viral myocarditis together with cell adhesion molecules (CAMs) were significantly overexpressed in tumor specimens. Contrarily, gene sets corresponding to the cell cycle, DNA replication, DNA repair and RNA transport were enriched in CUTO and HCC-78 cell lines. Therefore, these results highlight the major biological differences between tumor samples and cell lines and can be summarized in cell cycle, DNA repair and inflammatory signatures.

### 3.5 Differences between *ROS1*+ NSCLC cell lines encompass EMT- and Myc- related hallmarks

**Figure 4:**
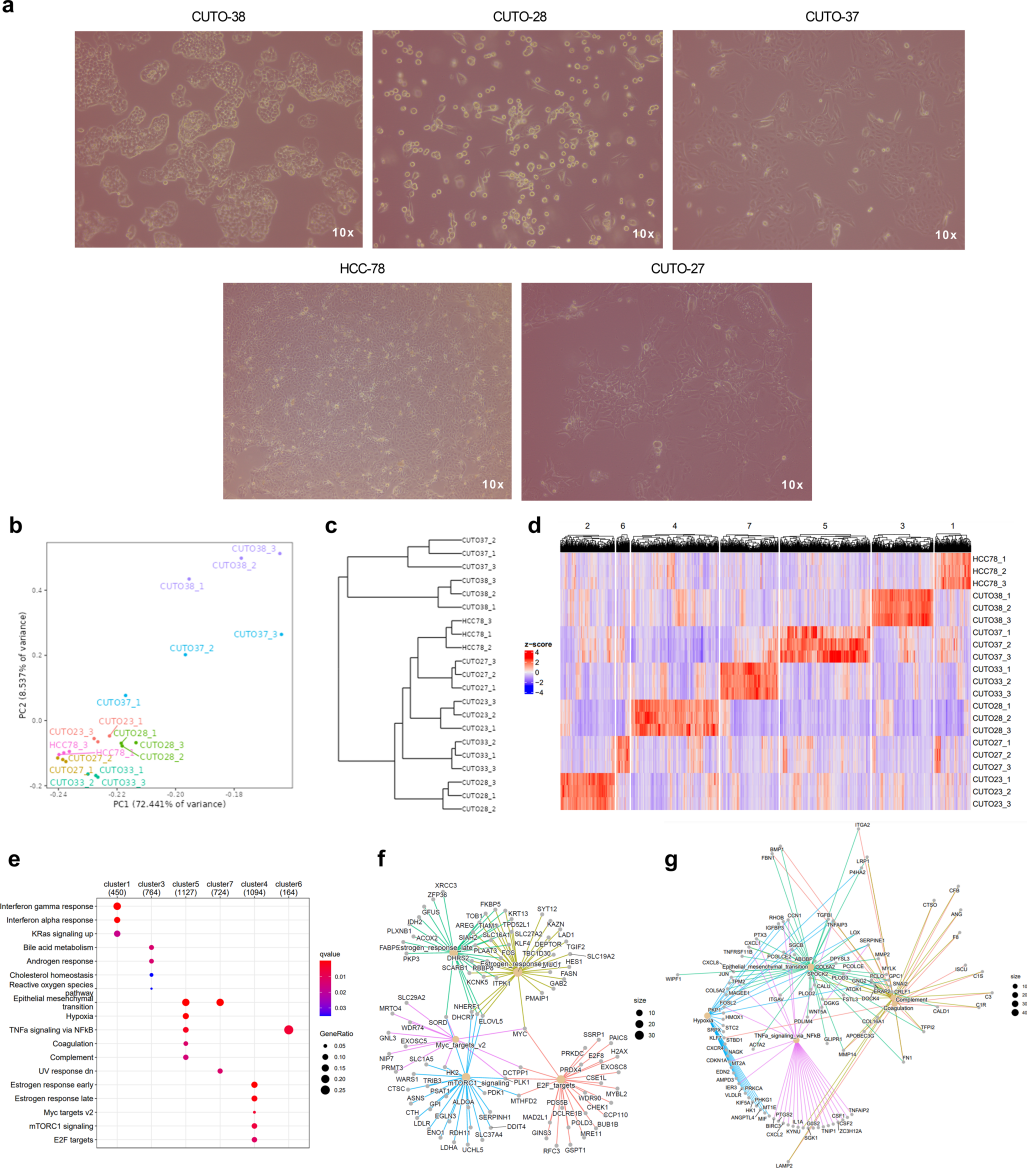
Transcriptomic traits of *ROS1*+ patient-derived NSCLC cell lines. **(A)** Phenotypes of some of the cell lines characterized in the study. Phase contrast microscopy images taken at 10X magnification. **(B)** Principal component plot. **(C)** Dendrogram and **(D)** clustergram reflecting the differences across cell lines. **(E)** GSEA indicating the significantly enriched hallmarks in each cell line. **(F)** Enriched hallmarks in CUTO-28 and **(G)** CUTO-37 cell lines.

The subsequent exploratory analysis focused exclusively on *ROS1*+ NSCLC patient-derived cell lines. As illustrated in **Figure 4A**, these cell lines exhibit a remarkable phenotypic heterogeneity. Their morphology and colony formation do not adhere to a single pattern, leading us to hypothesize that such differences might be reflected at the transcriptomic level. Despite the limited sample size, the differential gene expression analysis unveiled intriguing features, as depicted in the principal component analysis (PCA) shown in **Figure 4B**. The majority of cell lines clustered together, with a clear separation along the first principal component, which accounted for 72.44% of the variance. CUTO-37 and CUTO-38 were the two cell lines that clustered more distinctly from the rest. This observation is further supported by the dendrogram presented in **Figure 4C**.

Next, we identified similar expression patterns per each cell line through a k-means sample clustering. Assuming that each cell line constitutes a separate group and given that the biological replicates clustered together in each line, a K=7 was chosen. The resulting clustergram is shown in **Figure 4D**, and the following clusters are: 1: HCC-78 (450 genes), 2: CUTO-23 (314 genes), 3: CUTO-38 (764 genes), 4: CUTO-28 (1094 genes), 5: CUTO-37 (1127 genes), 6: CUTO-27 (164 genes) and 7: CUTO-33 (724 genes). The subsequent GSEA performed in order to identify the significantly enriched hallmarks in each cluster is shown in **Figure 4E**. HCC-78 transcriptome was enriched in KRas-mediated signaling and interferon responses, CUTO-38 cells showed an enrichment in cholesterol and reactive oxygen species (ROS) together with androgen response and bile acid metabolism. CUTO-37 cells were enriched in the EMT, hypoxia and TNFα signaling via NF-ƙB, among other hallmarks. CUTO-33 cells were EMT- and UV response-enriched hallmarks. CUTO-28 cells showed an enrichment in estrogen responses, Myc targets, mTORC1 signaling and E2F targets hallmarks. Finally, CUTO-27 depicted an upregulation in TNFα signaling via NF-ƙB hallmark. No significant hallmarks were identified in CUTO-23 cells. The networks of the GSEA hallmarks identified in the two most distant cell lines according to the dendrogram, CUTO-28 and CUTO-37 are represented in **Figure 4F** (CUTO-28) and **Figure 4G** (CUTO-37) respectively.

### 3.6 The *ROS1* fusion partner genes modulate modestly the tumor transcriptome

**Figure 5:**
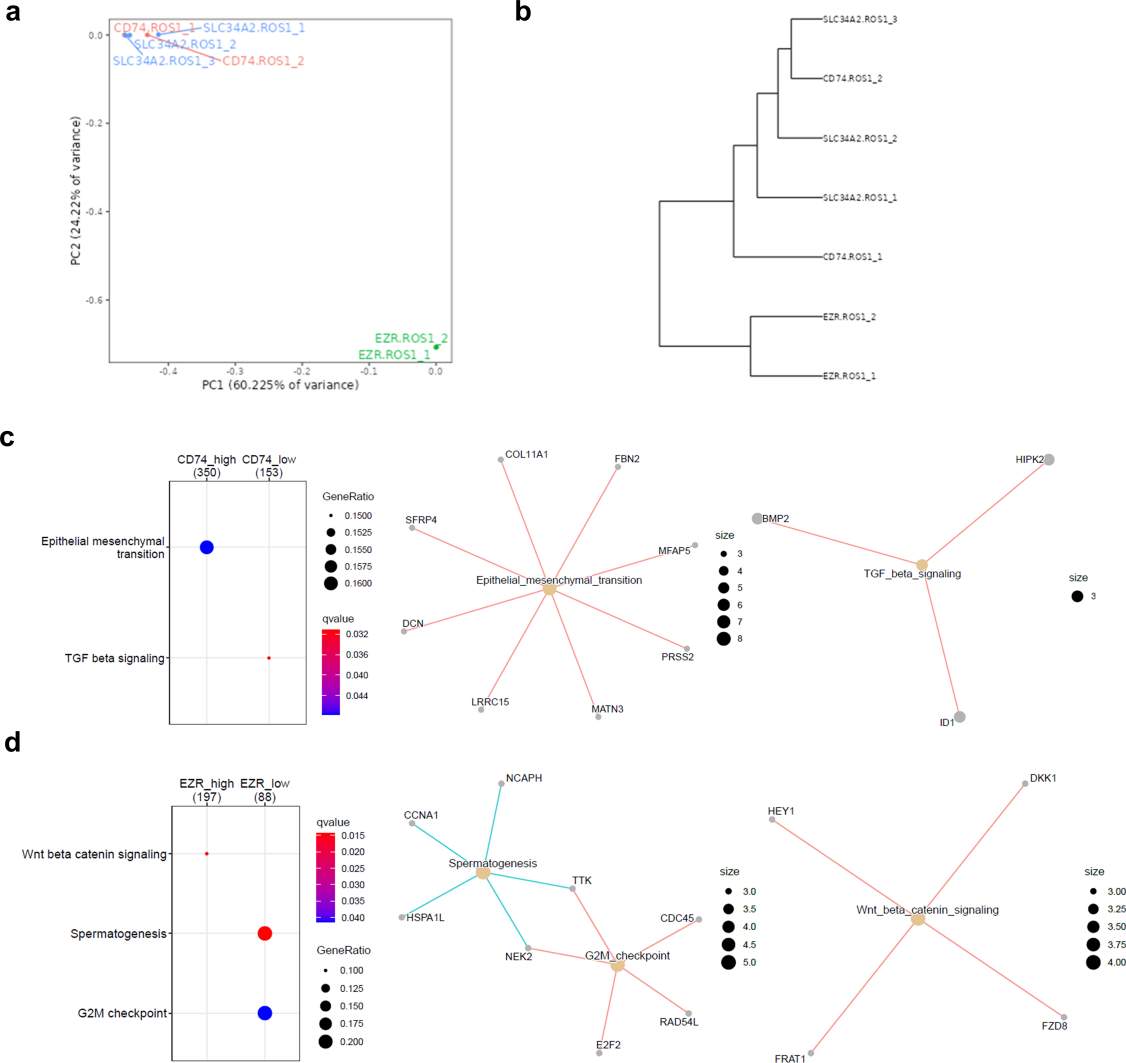
Impact of the *ROS1* fusion partner in the tumor transcriptome. **(A)** Principal component analysis (PCA) of *CD74*-, *SLC34A2*- and *EZR*-*ROS1* tumor samples. **(B)** Clustergram of the cell lines based on their transcriptomic traits **(C)** GSEA depicting the enriched hallmarks in *CD74*-*ROS1* and **(D)** *EZR*-*ROS1* NSCLC samples.

The molecular subtypes defined by the ROS1 fusion partner genes have been shown to influence downstream signaling due to the different subcellular localizations of the resulting fusion proteins (25). Therefore, we hypothesized that the differential activation of signaling pathways might be reflected at the gene expression level. Three out of the four *ROS1*-rearranged tumor subtypes were included in the analysis, as there were at least two independent samples per fusion type (*CD74*-*ROS1*, *SLC34A2*-*ROS1*, and *EZR*-*ROS1*). The PCA plot containing PC1 and PC2 indicates that *EZR*-*ROS1* tumors cluster remarkably further from *CD74*- and *SLC34A2*-*ROS1* tumor specimens, as shown in **Figure 5A**. The complementary dendrogram showcases the similarity between *CD74*- and *SLC34A2*-*ROS1* tumors (**Figure 5B**).

Next, a DEG analysis was conducted to unveil the distinctive traits of each *ROS1* rearrangement type, done through pairwise comparisons. The cut-off conditions were log_2_(fold change |2|) > and FDR < 0.05. The obtained volcano plots and heatmaps per each rearrangement are shown in **Supplementary Figure 2B**, for the *CD74-ROS1* signature, 2c for *SLC34A2-ROS1* signature and 2d for *EZR-ROS1* signature respectively. The functional annotation of the DEGs in each subtype revealed a significant upregulation of *CD74-ROS1* tumors in EMT hallmark and a downregulation of TGF-β signaling (**Figure 5C**). In addition, *EZR-ROS1* tumor specimens were characterized by an enrichment in β-catenin signaling and the downregulation of a G2M checkpoint hallmark (**Figure 5D**). It is worth noting that no significant enrichment was observed in the *SLC34A2-ROS1* subtype.

Finally, the correlation between *ROS1* expression and selected oncogenes of interest was determined for the *CD74*-*ROS1*, *SLC34A2*-*ROS1*, and *EZR*-*ROS1* subtypes (**Supplementary Figure 2**). Notably, *MET* (Spearman correlation coefficient = 0.964), *BRAF* (0.963), *MYC* (0.714), and *EGFR* (0.39) exhibited positive correlations with ROS1 expression. Conversely, the expression of transcription factors known to drive epithelial-mesenchymal transition (EMT), such as *SNAI1* (-0.678) and *TWIST1* (-0.785), negatively correlated with *ROS1* expression. However, these correlations did not reach statistical significance, which might be attributed to the limited sample size in each group.

### 3.7 The connexin-encoding *GJB2* is expressed in a *CD74*- and *CLTC*-*ROS1*-dependent manner

**Figure 6:**
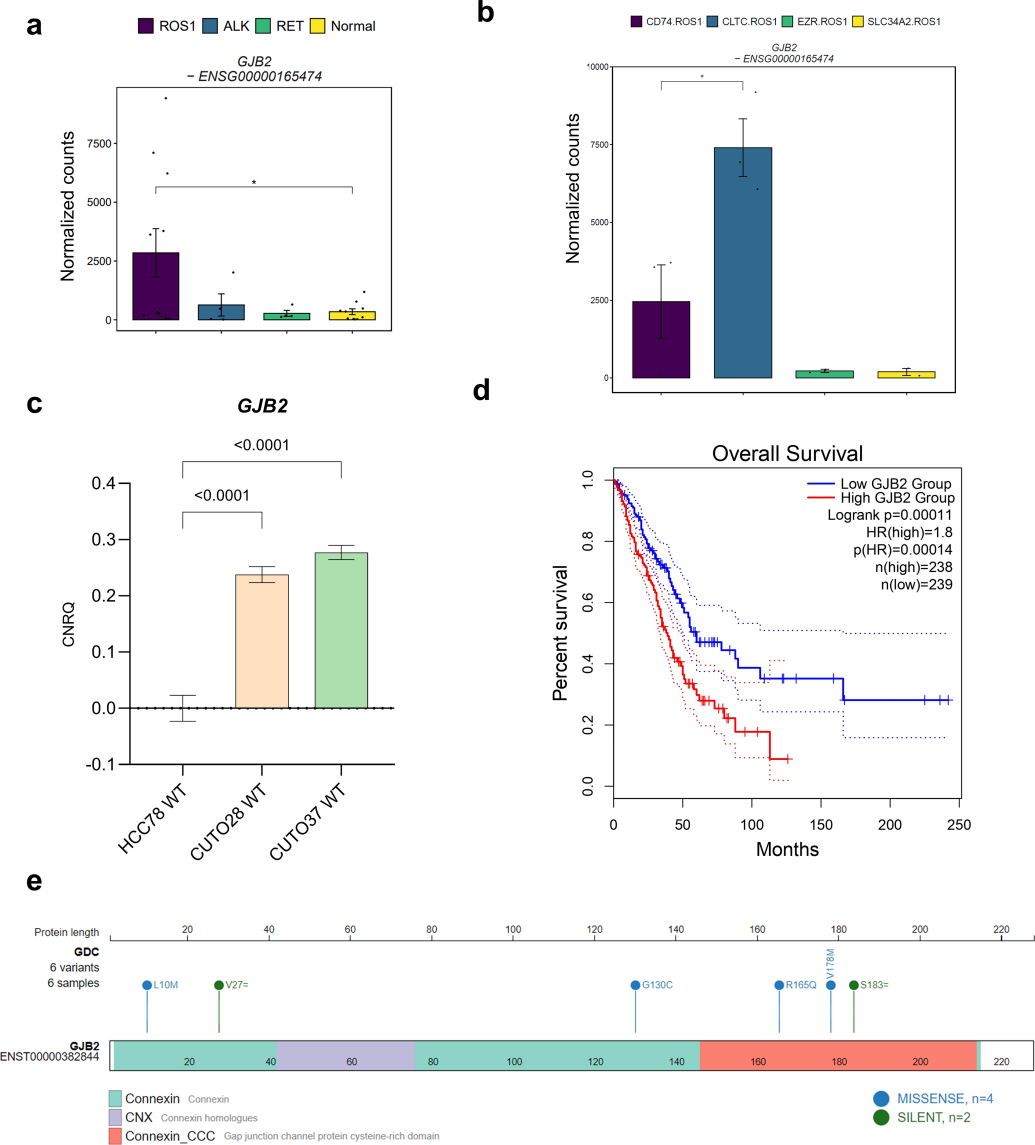
The role of *GJB2* expression in *ROS1*+ NSCLC. **(A)** *GJB2* expression levels across oncogene-driven NSCLC and normal lung tissue. **(B)** *GJB2* expression across different *ROS1* rearrangements in tumor specimens. **(C)** *GJB2* mRNA levels in HCC-78, CUTO-28 and CUTO-37 cell lines expressed as calibrated and normalized relative mRNA quantity (CNRQ) ± SEM. A 1-way ANOVA test was performed considering a Bonferroni correction and a chosen α = 0.05. **(D)** Kaplan-Meier survival plot of The Cancer Genome Atlas (TCGA) lung adenocarcinoma (LUAD) cohort divided in *GJB2*-high or low expression levels. **(E)** *GJB2* single-nucleotide variants identified in LUAD patients.

Based on our hypothesis, we proceeded to evaluate *GJB2* expression as a potential prognostic marker for ROS1-rearranged NSCLC, considering its upregulation compared to *ALK*+ and *RET*+ tumors, as well as normal adjacent lung tissue. Statistical significance was achieved only when compared to normal lung tissue (p = 0.042) (**Figure 6A**). This observation might be attributed to the fact that *GJB2* overexpression occurs only in specific *ROS1* fusion subtypes. **Figure 6B** illustrates the normalized *GJB2* counts across the investigated *ROS1* rearrangement types, revealing that *GJB2* expression was present only in *CD74*-*ROS1* and *CLTC*-*ROS1* samples, with a significant upregulation in the latter subtype (p = 0.016). These findings were validated through RT-qPCR in our cell line models, where transcript levels are expressed as calibrated and normalized relative mRNA quantities (CNRQ) ± SEM (**Figure 6C**). *GJB2* expression was not detected in HCC-78 cells harboring the *SLC34A2*-*ROS1* fusion, in contrast to CUTO-28 (*TPM3*-*ROS1*) and CUTO-37 (*CD74*-*ROS1*) cell lines, which showed significant *GJB2* expression (p < 0.0001 in both comparisons). Consequently, the higher levels of *GJB2* transcript in CUTO-28 and CUTO-37 lines suggest that *GJB2* is actively expressed by tumor cells and that its expression varies depending on the specific *ROS1* fusion type.

Moreover, we evaluated the prognostic significance of high *GJB2* expression in the lung adenocarcinoma (LUAD) cohort from The Cancer Genome Atlas (TCGA) (**Figure 6D**). LUAD patients with *GJB2* overexpression exhibited a poorer prognosis compared to those in the low-*GJB2* expression group (Hazard ratio for high *GJB2* expression = 1.8; p-value for high *GJB2* expression < 0.001). Next, we validated this finding in an independent LUAD patient cohort that combined several studies. As shown in **Supplementary Figure 4**, the results were concordant with the TCGA cohort. Taking these observations into account, *GJB2* expression might explain the variability among *ROS1*+ NSCLC patient disease outcomes. As reported by Liu et al., the potential mechanism behind the pro-tumorigenic role of high *GJB2* expression might be the upregulation of the SPP1 signaling pathway (24). Furthermore, we mapped the reported *GJB2* variants within the same TCGA LUAD cohort onto the gene structure, as illustrated in **Figure 6E**, to identify potential gain-of-function mutations. Among the variants, four were missense mutations: L10M and G130C, located in the connexin domain; and R165Q and V178M, present in the cysteine-rich connexin domain of GJB2. Interestingly, only the V178M variant has been clinically described in the literature, reported as a pathogenic mutation identified in patients with autosomal-recessive hearing loss (38). This suggests that V178M is unlikely to be a gain-of-function mutation in the context of hearing loss. However, the impact of the GJB2 V178M variant on tumor cell biology remains to be elucidated.

### 3.8 Gene co-expression analysis proposes TFDP1 as master transcription factor in *ROS1*+ NSCLC

**Figure 7:**
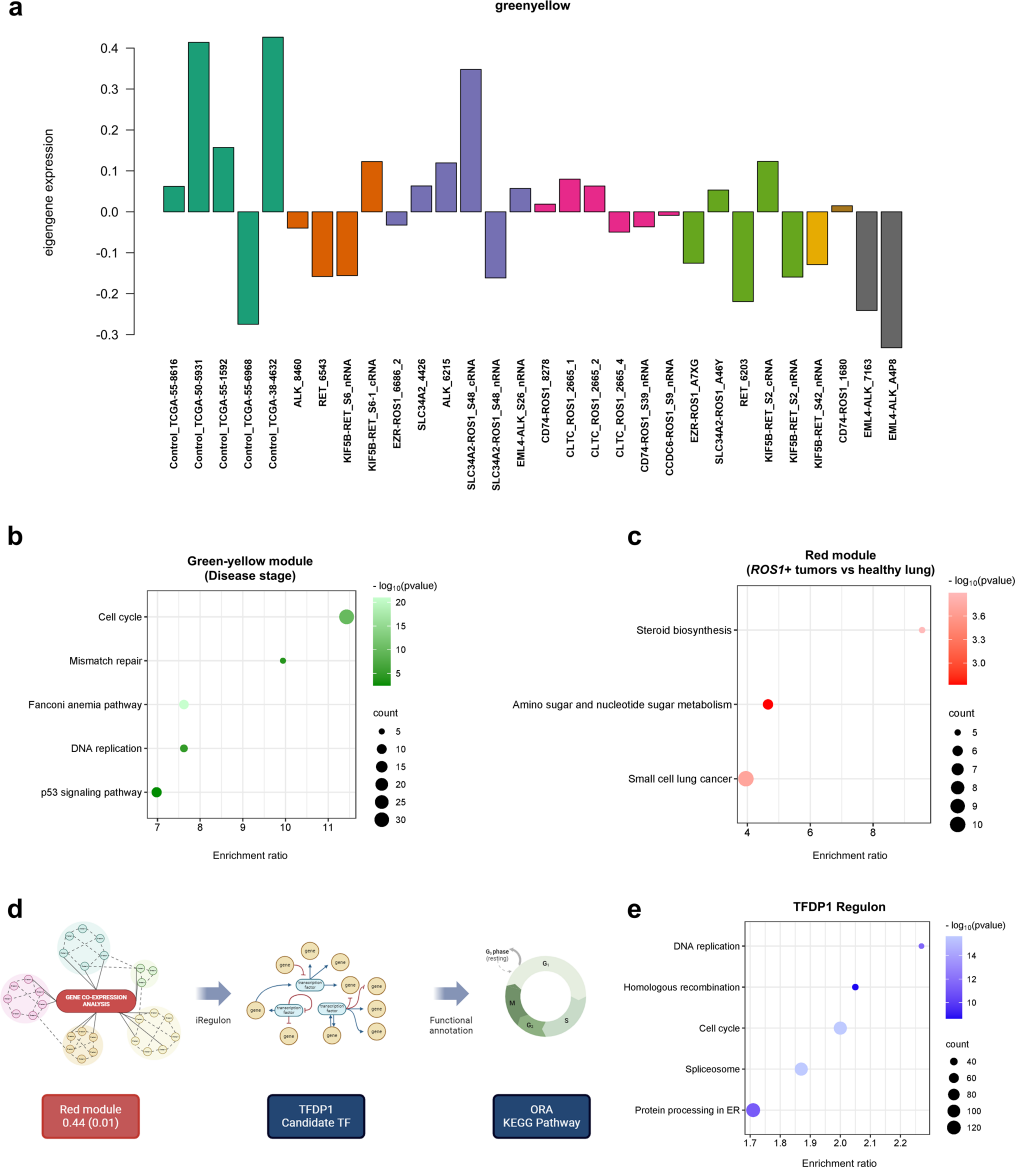
Gene co-expression analysis. **(A)** Eigengene expression patterns of the green-yellow module across samples. **(B)** ORA comprising the green-yellow gene module and **(C)** red module. **(D)** Workflow performed to identify key transcription factors (TFs) that regulate the expression of genes in a given co-expression module. **(E)** ORA performed with the TFDP1 regulon.

Unsupervised methods, such as gene co-expression analysis, enable the identification of gene clusters with positively or negatively correlated expression patterns, independent of sample category or experimental condition. In this study, we identified 18 gene clusters, each characterized by a correlation coefficient and a p-value associated with a specific trait or phenotype. **Supplementary Figure 5** illustrates the resulting matrix, with columns representing the traits of the tumor samples included in the analysis. The strongest correlation was observed for the green-yellow cluster (coefficient = -0.48, p = 0.007) with respect to disease stage, suggesting that the expression patterns of genes within this cluster collectively explain a portion of the variability attributed to the disease stage of the samples. The observed eigengene expression patterns of the green-yellow module across samples (**Figure 7A**) highlight the variability in the direction and magnitude of gene expression among different sample types. These discrepancies can be attributed to the moderate correlation coefficient of the module, indicating that while the genes within the cluster share a common expression pattern, other factors may also influence their expression. The functional annotation of this gene cluster through over-representation analysis (ORA) revealed the involvement of genes in critical cellular processes such as cell cycle, DNA repair and replication, and the P53 signaling pathway (**Figure 7B**). These findings suggest that the disease stage of tumor samples can be partially explained by the coordinated expression of genes regulating these essential pathways.

The following module which showed moderate correlation between the genes is the red one (0.44, p=0.01). The trait that defines this gene cluster is the comparison between *ROS1*+ tumor specimens and normal or healthy lung samples. When carrying out ORA, the steroid biosynthesis, amino sugar and nucleotide sugar metabolism together with the small cell lung cancer KEGG pathways were identified (**Figure 7C**). Finally, with the aim to unveil master regulatory transcription factors that orchestrate the *ROS1*+ NSCLC transcriptome, an iRegulon-based analysis upon the red module was performed followed by an ORA annotation (**Figure 7D**). The top-ranked transcription factor (TF) for the red gene module was TFDP1. Considering only the target genes of the aforementioned TF, ORA was performed (**Figure 7E**) and it unveiled that DNA replication, homologous recombination, cell cycle, spliceosome and protein processing in ER were the significantly over-represented KEGG pathways in the TFDP1 regulon.

## 4 Discussion

The biology of oncogene-addicted lung neoplasms has been heterogeneously approached within the last decades. Partially due to differences in the prevalence of each molecular subtype, restricting access to patient-derived samples. In consequence, study of *ROS1*-, *ALK*- and *RET*- rearranged tumors remains modest beyond the kinase domain of their oncogenic fusion proteins. To unveil distinctive traits between oncogene-addicted lung adenocarcinomas, particularly between rare subtypes like *ROS1*+ NSCLC, aspects such as tumor transcriptome profiling might be enlightening. Although existing transcriptome studies lack the necessary depth and specificity required for a comprehensive characterization of *ROS1*-driven NSCLC, we hypothesized that reanalysis based on combined data can yield a more representative characterization of this tumor type (39–41).

Our findings point towards a relevant contribution of the nucleotide synthesis and cell adhesion pathways to the proliferation of *ROS1*+ lung adenocarcinomas. The highest overexpressed gene in *ROS1*+ tumor specimens and cell lines, *ISL1* provides new insights about this molecular subtype. This gene encodes a LIM homeodomain transcription factor which regulates insulin expression during homeostasis. Moreover, its pathological role in tumorigenesis has been reported through the regulation of cyclin D1 and c-Myc genes in neuroblastoma, gastric cancer and non-Hodgkin lymphoma (42–44). *ISL1* has been recently reported to mediate lung morphogenesis, particularly, the adequate branching of the respiratory epithelium through the Sonic hedgehog (Shh) signaling pathway (45). In addition, *ISL1* is known to regulate processes like epithelial cell differentiation, adhesion and migration in NSCLC (46, 47). Importantly, Shh mediates processes like EMT, therefore a dysregulation of *ISL1* expression might enhance tumorigenesis via the acquisition of a mesenchymal phenotype of neoplastic cells. Li et al. provided evidence supporting this hypothesis by demonstrating that triptolide-mediated inhibition of the Sonic hedgehog (Shh) pathway could reverse chemoresistance in NSCLC cells (48). Regarding the upregulated cell adhesion signature in *ROS1*+ NSCLC, the gene encoding Ras interacting protein 1 (*RASIP1*) was found to be significantly overexpressed. RASIP1 plays a crucial role in the integrin activation process, which is essential for cell migration (49). Therefore, pharmacological inhibition of RASIP1 or its associated pathways might represent a potential strategy to prevent or reduce the development of metastasis in *ROS1*+ NSCLC.

Additionally, the significantly increased levels of *NOTCH1* in *RET*+ tumors might partially explain the higher rates of extrathoracic metastases in *RET*+ compared to *ROS1*+ NSCLC patients. The potential underlying mechanism might be the epithelial-to-mesenchymal transition (EMT)-promoting role of the Notch pathway that enhances cell migration (19, 22, 50). As depicted by two studies in NSCLC, Notch-1 and Notch-3 respectively promote EGFR-TKI resistance and maintain a stem-like status. Remarkably, their pharmacological inhibition decreased cell growth and migration, autophagy and increased the cell apoptotic activity (18, 20, 21). Hence, the Notch pathway constitutes a crucial player regarding the regulation of EMT in NSCLC, becoming a drug target for metastatic disease.

It is known that higher *GJB2* expression levels in a subset of cancer-associated fibroblasts (CAFs) induce stromal tumor fibrosis, enhancing chemotherapy resistance in solid tumors (51). These observations were complemented by the significant positive correlation between *GJB2* expression and the tumor infiltration by CAFs (52). Specifically in LUAD, the promoter of *GJB2* was found to be hypomethylated, possibly leading to gene overexpression. Moreover, the functional annotation of DEGs in *GJB2*-overexpressing LUAD revealed an enrichment of the PI3K/AKT and ECM-receptor interaction KEGG pathways (53). However, the functional characterization of *GJB2* missense variants identified in patients is currently lacking, limiting our ability to draw definitive conclusions regarding their impact on gene function and disease pathogenesis. In parallel, the upregulation of *IL20RB* in *ROS1*+ NSCLC tumors and cell lines might constitute one of the mechanisms that mediate bone metastasis. According to a recent study performed in The Netherlands, around 1/3 of stage IV *ROS1*+ NSCLC patients present bone metastases at diagnosis (54). IL20RB has been described to activate the JAK/STAT pathway upon stimulation with interleukin 19 (IL-19). In consequence, phosphorylated STAT3 translocates to the nucleus of tumor cells promoting the secretion of granulocyte-macrophage colony stimulating factor (GM-CSF), a cytokine that induces IL-19 synthesis in osteoclasts (33). Importantly, IL20RB neutralization with antibodies reduced the metastatic potential of tumor cells *in vivo*; therefore this becomes a relevant strategy to be evaluated in clinical trials in order to manage bone metastases in advanced stages.

The identification of the enriched systemic lupus erythematosus (SLE) signature in *ROS1*+ NSCLC might be *a priori* a puzzling finding. SLE is an autoimmune disease characterized by a dysregulation of cytokines, T cells, B cells and macrophages (55). However, this signature has been previously identified in lung cancer with a higher incidence in the adenocarcinoma histological subtype and non-smoker women (56). Although the causal explanation of this findings is not fully understood, the manifestations of the SLE signature are consistent with the higher risk of thromboembolic events reported in *ROS1*+ NSCLC patients (57–59). Mechanistically, SLE-induced coagulopathy is caused by autoantibodies which target endothelial cells. The resulting damage triggers the coagulation cascade (60). Therefore, in the context of *ROS1*+ NSCLC, the expression of a SLE gene signature might be mediated by cell adhesion molecules; another important gene set identified in this study (61, 62). The potential aberrant expression of cell adhesion molecules like integrins and gap junction proteins could initiate the coagulation cascade, explaining the increased susceptibility of thromboembolic events observed in patients.

Importantly, the main limitation of this study is the low sample size of *ROS1-*, *ALK-* and *RET*-rearranged tumors, which is explained by the low prevalence of each tumor subtype. The limited number of tumor specimens and cell lines included in our analysis may affect the generalizability of our findings, necessitating further validation using additional patient-derived samples and experimental models. Moreover, the patients involved in the study received various treatment regimens, which likely impacted gene expression profiles, introducing another potential limitation of our study. However, the identified signatures emerged from analyzing tumor samples and patient-derived cell lines independently collected and sequenced, increasing the robustness of the presented evidence. By employing RT-qPCR, we validated the expression of *IL20RB* in our *ROS1*+ cell lines as well as the fusion-specific *GJB2* expression pattern. Consequently, we confirm the validity of such findings in independent samples and using an orthogonal method to NGS like RT-qPCR. In addition, complementing the dataset with patient-derived cell lines provides two main advantages. First, the confirmation of the observations in tumor samples, a biologically comprehensive sample type. Second, a detailed assessment of the cell line transcriptomes unveiled converging gene expression patterns with tumor samples, which can be summarized in the enrichment of cell adhesion molecules and nucleotide synthesis signatures. The shared traits offer a new insight about the most representative features of cell lines’ tumor physiology, shown by a dependency on *ISL1* expression which links its activity with glucose metabolism and the acquisition of a mesenchymal phenotype.

As seen in our study, differences were noticed between tumor samples and cell lines, reflected by the more than 6000 significantly DEGs. Among them, key oncogenes like *MYC* and *MET* were found to be upregulated in cell lines; possibly as a result of the spontaneous immortalization of cell lines. Moreover, the observed cluster of overexpressed genes on chromosome 7, in line with previously reported copy number gains of 7p22.3 in cell lines, must be considered when extrapolating results obtained *in vitro*. Because this region is rich in oncogenes, differences should be closely regarded when designing experiments involving pathways in which these genes participate (16, 37). It is also worth noting that HCC-78 cells clustered among CUTO lines, indicating that their transcriptomes are very similar despite being cell lines established in different time points. Nevertheless, the phenotypical and transcriptomic variability among *ROS1*+ patient-derived cell lines supports the heterogeneous *ROS1*+ NSCLC patient outcomes in the clinical setting, reinforcing them as valuable experimental models.

The mild impact of different *ROS1* fusion partner genes contrasts with the work by Neel et al. They demonstrated SLC34A2-ROS1 and SDC4-ROS1 fusions strongly activate the MAPK pathway, which was not seen in CD74-ROS1, due to their differential subcellular localization. The discordance with our results might be explained by three reasons. First, the included data did not include determination of phosphorylated Erk 1/2 protein through immunoblotting, which would have been the most specific method to determine the activation levels of the MAPK pathway. Second, the overlap between the target genes of the MAPK pathway and other ROS1 downstream signaling pathways, such as the JAK/STAT or mTOR/AKT, might mask the differential MAPK pathway activation levels from a transcriptome perspective. Third, Neel et al. also mentioned that a shorter CD74-ROS1 isoform can localize in endosomes and plasma membrane instead of the ER, and as such activate the MAPK pathway as well (25).

Nonetheless, the *GJB2* overexpression found in *CLTC*-*ROS1* and *CD74*-*ROS1* subtypes, might have implications in disease progression. First because *GJB2*, expressed by tumor cells and cancer-associated fibroblasts (CAFs), contributes to ECM remodeling and activates the SPP1/PI3K/AKT signaling pathway in lung adenocarcinoma (24, 63). Second, due to the pro-metastatic role of *KRT16*, which was found to be upregulated in the same two patient subsets. (**Supplementary Figure 6**). This cytokeratin upregulates the synthesis of vimentin in lung cancer cells (64). Consequently, this hypothesis is concordant with the increased likelihood of *CD74-ROS1*+ NSCLC patients to develop brain metastases (65). On top of that, Wang et al. identified that *CD74*-*ROS1*+ bone metastatic NSCLC cells secreted CCL5 through STAT3 activation to recruit macrophages. In this interaction, the tumor-promoting M2 macrophages stimulate tumor cells and induce EMT via TGF-β pathway stimulation (66). These results align with the upregulated EMT signature that we report in *CD74*-*ROS1* specimens. Similarly, the downregulation of the TGF-β pathway that we found in tumor specimens can be explained by the lack of interaction between tumor cells and the bone niche in the samples that we analyzed.

Applying gene co-expression analysis in the tumor samples lead to the detection of the cell cycle and amino sugar and nucleotide sugar metabolism pathway over representation within the *ROS1*+ lung adenocarcinoma subtype. This approach allowed an unsupervised characterization of samples aimed to identify functionally related genes (67). The confirmation of these observations in additional samples, experimental models and independent cohorts might open the door to find new actionable targets in a *ROS1*-specific manner. In parallel, this approach combined with the iRegulon tool pointed towards TFDP1, a transcription factor known to interact with E2F, another essential regulator whose targets were significantly enriched in CUTO-28 cells. Moreover, *TFDP1* amplification has been described in lung cancer and esophageal squamous cell carcinomas (68). It is important to note that an activation of the TFDP1/E2F1 axis in lung cancer results in the attenuation of the p53 pathway, mediated by COMMD9 (69). Thus, this transcription factor represents a novel research object holding therapeutic potential.

Focused efforts concerning *ROS1*+ NSCLC patients are currently oriented towards refining the treatment scheme upon improved patient stratification, overcoming drug resistance and understanding the disease risk factors (70). Our study contributes to two of these aspects. First, treatment-wise, combination strategies of TKIs and monoclonal antibodies targeting IL20RB might be beneficial to treat bone metastases. Second, further functional studies are required to confirm the role of the nucleotide synthesis pathway in *ROS1*+ NSCLC, its targeting might enhance the sensitivity towards checkpoint inhibitors as reported by Wu et al. (71) Besides, the inhibition of nucleotide synthesis through mTORC1/IMPDH targeting is known to induce replication stress, ultimately resulting in apoptosis (72). Thus, exploring combinations of TKIs and IMPDH inhibitors could be useful to address heavily pre-treated cases. Third, *GJB2* expression could be employed as a prognostic biomarker. Collectively, such observations have the potential to enhance the tailoring of therapies to different patient subsets and help to predict disease outcomes.

## 5 Conclusion

In the present study we perform an in-depth characterization of *ROS1*+ NSCLC using two complementary approaches: differential gene expression and gene co-expression analysis. Our results point towards *IL20RB*, the nucleotide synthesis and cell adhesion pathways as specific signatures compared to *ALK*+ and *RET*+ tumors. Importantly, they constitute targetable alterations which could be co-inhibited together with ROS1. Moreover, we report differences in oncogene expression such as *MYC*, *MET* and *BRAF* between *ROS1*+ tumor samples and cell lines, which should be taken into account when interpreting *in vitro* experiments. Finally, we propose ISL1 and TFDP1 as candidate transcription factors that complement the oncogenic dependencies of *ROS1*+ NSCLC through cyclin D1, c-Myc and the Sonic hedgehog (Shh) pathway. Furthermore, the identification of the enriched systemic lupus erythematosus (SLE) signature might be related to the higher risk of thromboembolic events in *ROS1*+ NSCLC patients. In addition, *GJB2* was found overexpressed in *CD74*- and *CLTC*-*ROS1*+ tumor specimens and cell lines, which positively correlates with patients presenting a poor prognosis. Despite the limited sample size, the robustness of our evidences is supported by the independent validation of *IL20RB* and *GJB2* expression using RT-qPCR. Collectively, the present study broadens our understanding of the molecular alterations in *ROS1*+ NSCLC, paving the path towards novel therapeutic strategies.

## Data Availability

The original contributions presented in the study are included in the article/**Supplementary Material**. Further inquiries can be directed to the corresponding author.
