# Supplementary material for: Transcriptomic analysis of ROS1+ non-small cell lung cancer reveals an upregulation of nucleotide synthesis and cell adhesion pathways
Source: Front Oncol. 2024 Dec 16;14:1408697. doi: 10.3389/fonc.2024.1408697 (PMC11683107; doi:10.3389/fonc.2024.1408697)
Supplement: Supplementary file 1 [file DataSheet1.pdf]

Manuscript title: Transcriptomic analysis of ROS1+ non-small cell lung cancer reveals an upregulation of nucleotide synthesis and cell adhesion pathways

Manuscript ID: 1408697

Authors: Marc Terrones, Ken Op de Beeck, Guy Van Camp, Geert Vandeweyer and Ligia Mateiu

Supplementary figures

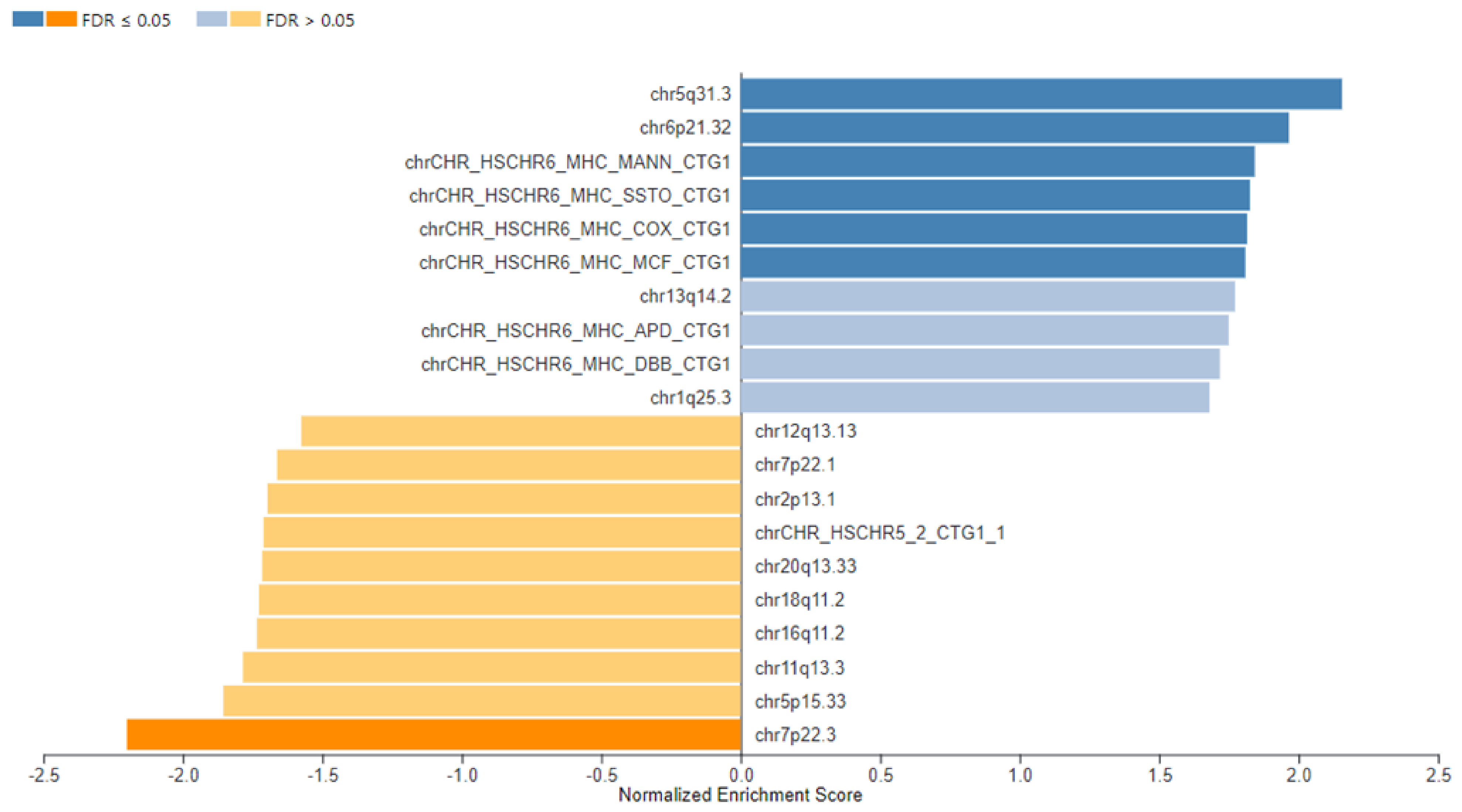

Supplementary figure 1 GSEA performed using the "chromosomalLocation" function with the significant DEGs resulting from the comparison between ROS1 tumor specimens and cell lines.

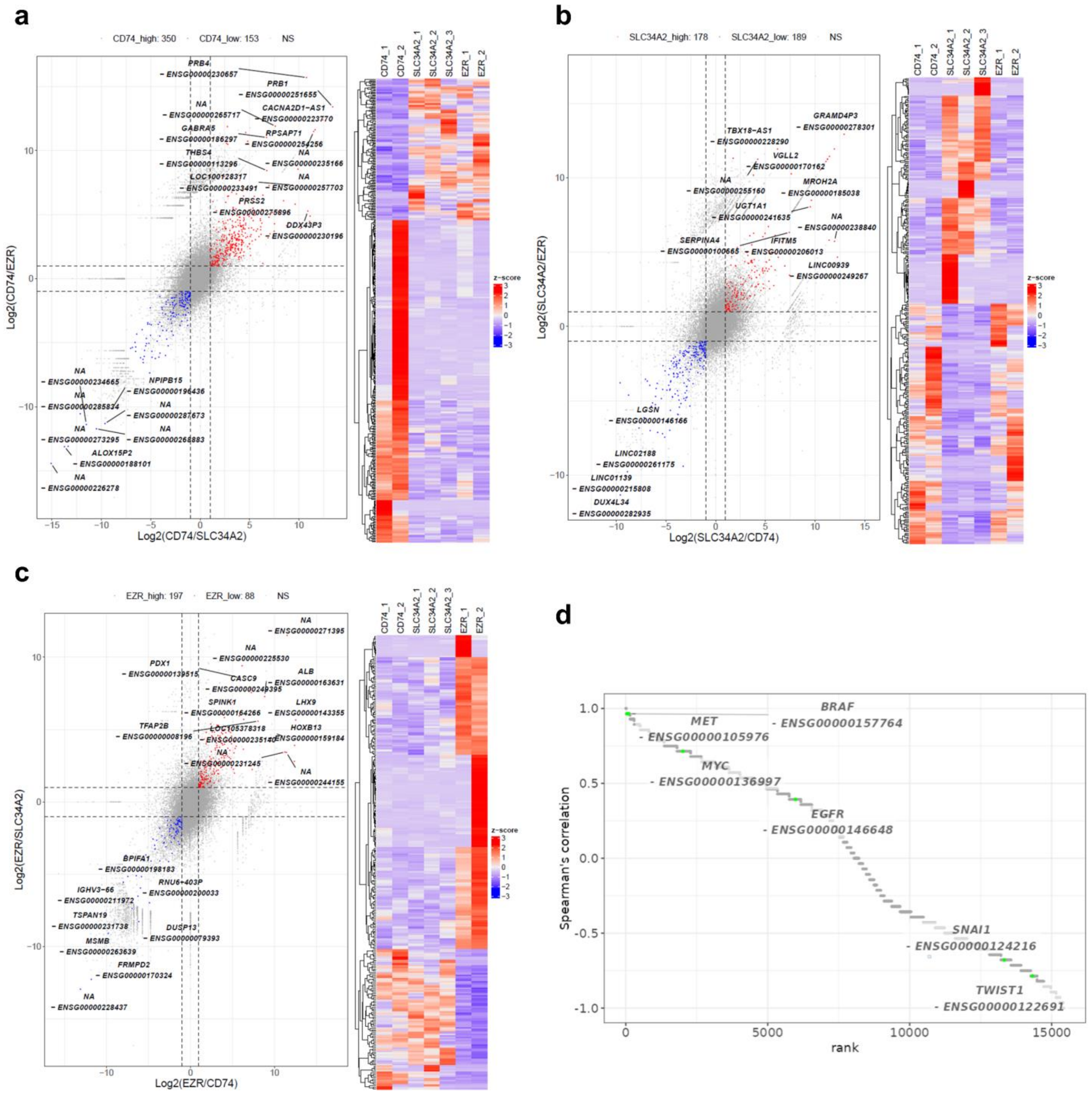

Supplementary figure 2 Impact of the *ROS1* fusion partner in the tumor transcriptome. Volcano plot and heatmap depicting **a** CD74-ROS1 **b** SLC34A2-ROS1 and **c** EZR-ROS1 signatures **d** Spearman correlation between ROS1 and oncogenes of interest across the three ROS1+ analyzed subtypes.

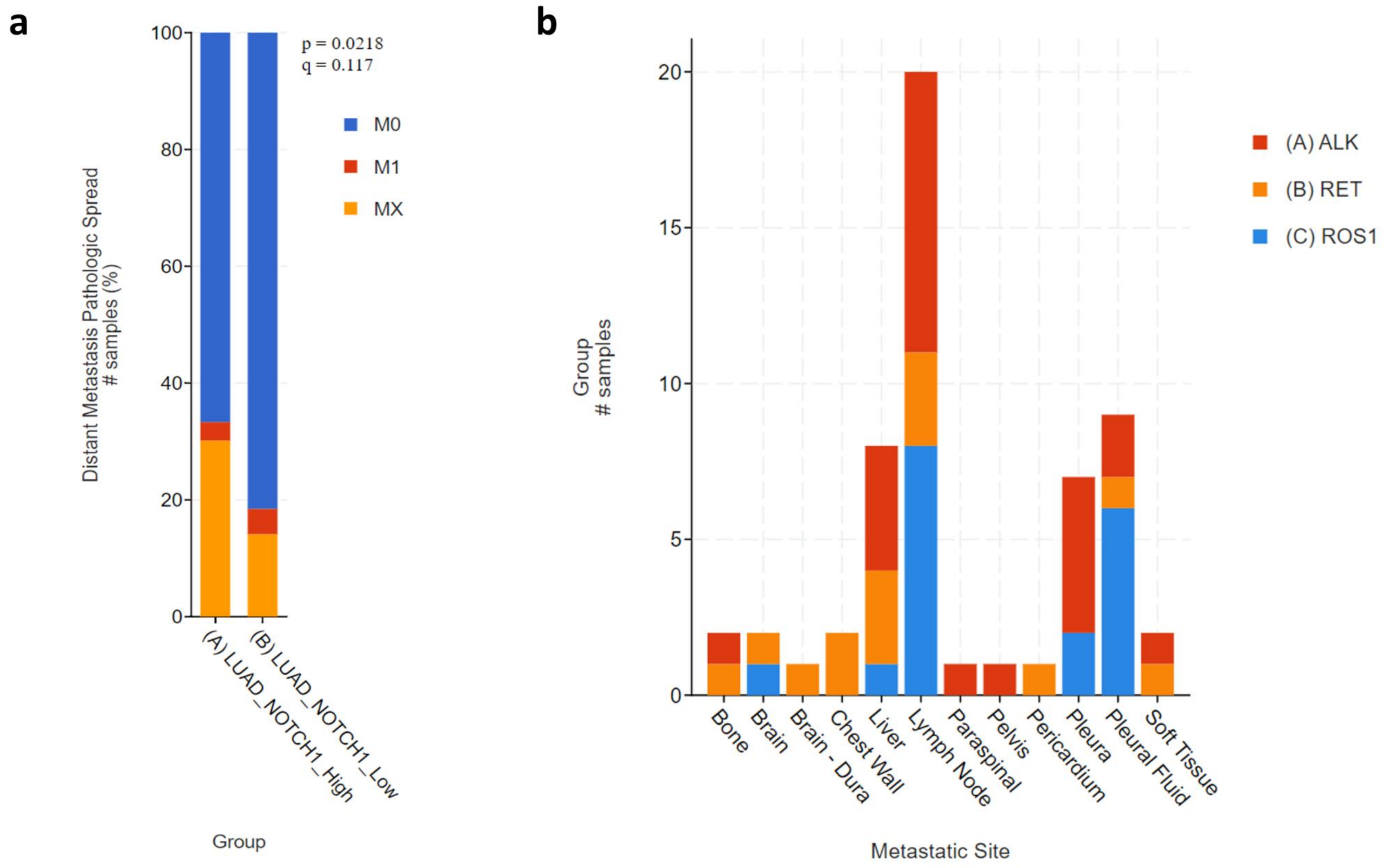

Supplementary figure 3 a Percentage of distant metastasis pathologic spread in LUAD patients presenting *NOTCH1* high or low expression respectively. **b** Distribution of metastatic sites across oncogene-driven LUAD patients.

***GJB2* expression in LUAD patients**
Independent cohort validation

The Kaplan-Meier plot integrates the data from the following studies:

Lung Adenocarcinoma (Broad, Cell 2012)
Lung Adenocarcinoma (CPTAC, Cell 2020)
Lung Adenocarcinoma (CPTAC, GDC)
Lung Adenocarcinoma (MSK, 2021)
Lung Adenocarcinoma (MSK, J Thorac Oncol 2020)
Lung Adenocarcinoma (MSK, NPJ Precision Oncology 2021)
Lung Adenocarcinoma (MSK, Science 2015)
Lung Adenocarcinoma (OncoSG, Nat Genet 2020)
Lung Cancer in Never Smokers (NCI, Nature Genetics 2021)

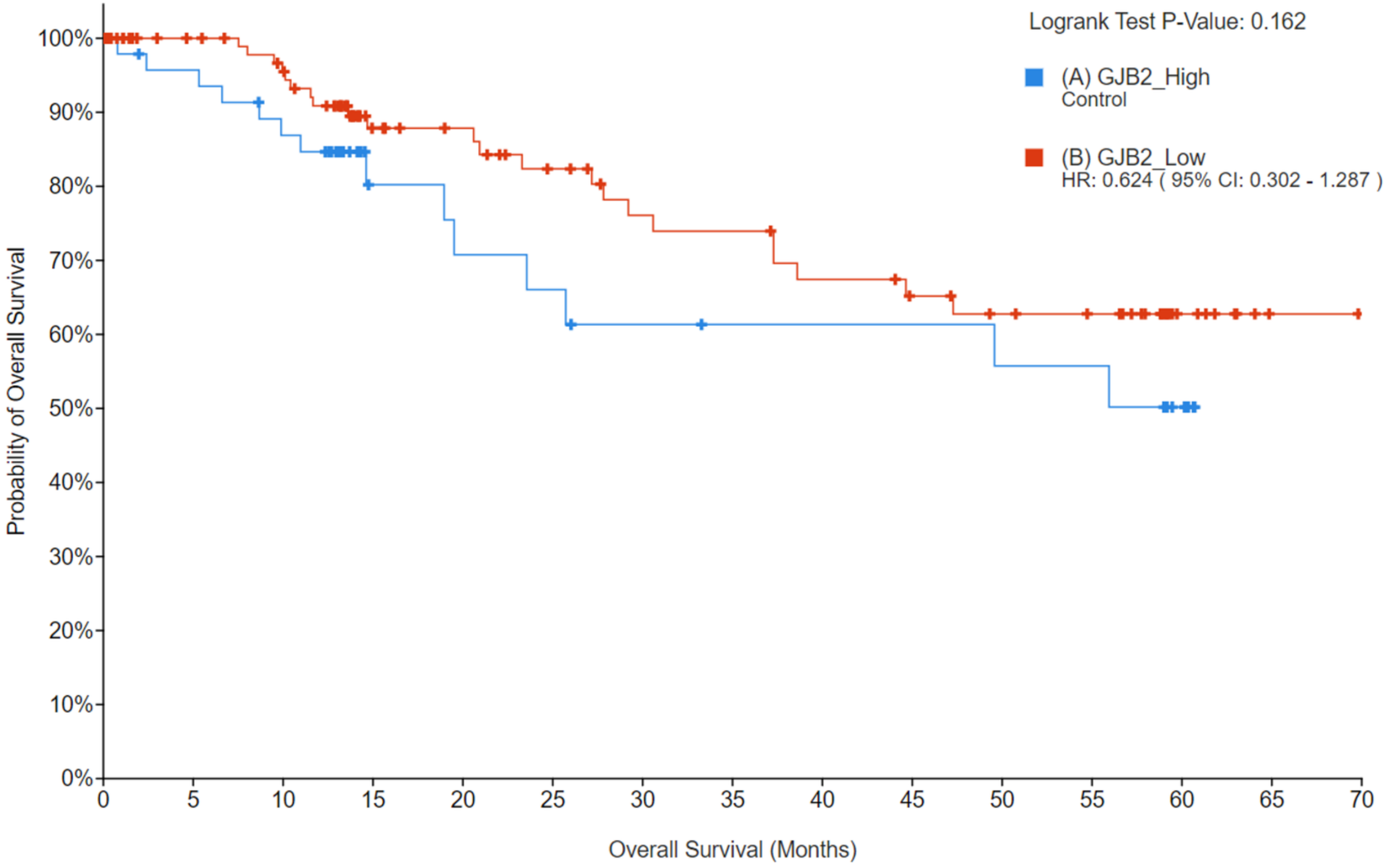

Number at risk (n)

(A) GJB2_High	49	44	39	17	15	14	12	11	11	11	10	10	6	0	0
(B) GJB2_Low	108	91	84	53	49	42	36	35	31	28	25	23	8	1	0

	Number of Cases, Total	Number of Events	Median Months Overall (95% CI)
(A) GJB2_High	**49**	**14**	**NA**
(B) GJB2_Low	**108**	**22**	**NA**

Supplementary figure 4 Patient survival analysis based on *GJB2* expression, TCGA-independent studies were used to validate our findings.

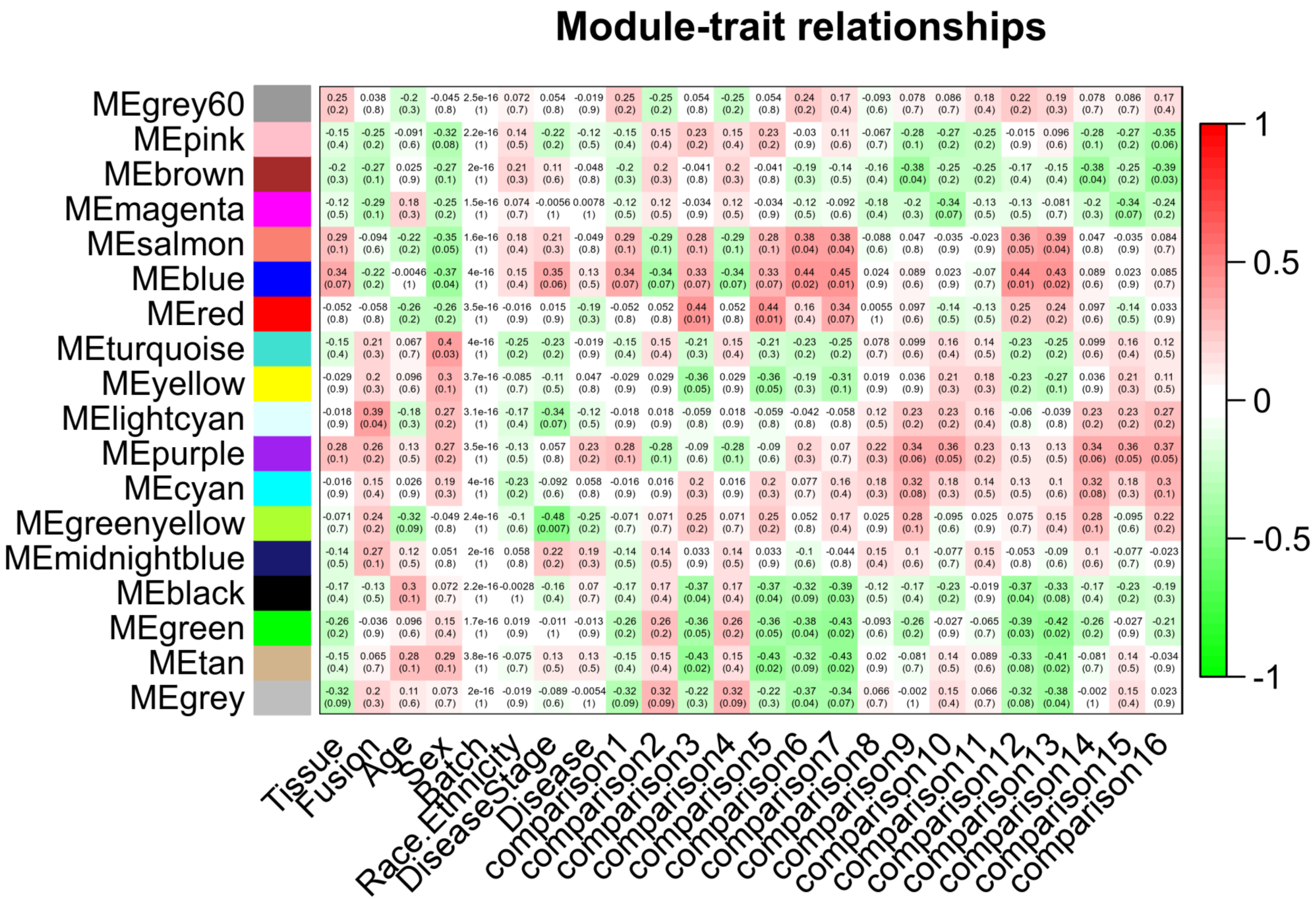

Supplementary figure 5 Gene co-expression correlation matrix between traits and modules. The samples included in each comparison are detailed in the supplementary excel table.

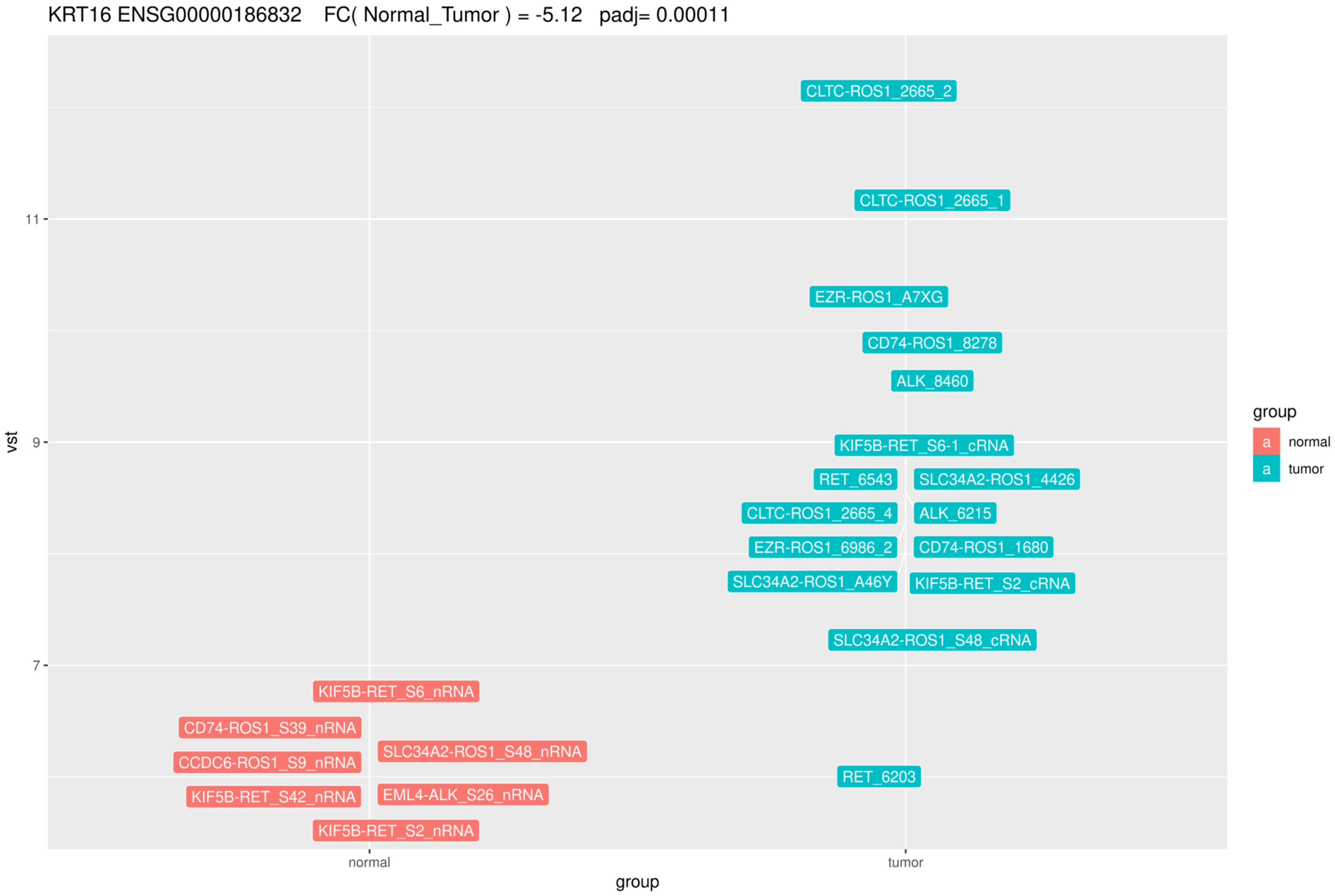

Supplementary figure 6 *KRT16* expression levels (variance stabilizing transformation) in tumor samples vs normal adjacent tissue.
